# Albumin Hydrogels Loading Bacteriophage PA57 as a Promising Platform for *Pseudomonas aeruginosa* Infection Management

**DOI:** 10.3390/gels12090817

**Published:** 2026-09-06

**Authors:** Inna Zharkova, Tatiana Ushakova, Yulia Tupikova, Oksana Gulyaeva, Vera Morozova, Yulia Kozlova, Nina Tikunova, Elena Dmitrienko

**Affiliations:** 1 Knorre Institute of Chemical Biology and Fundamental Medicine of the Siberian Branch of the Russian Academy of Sciences, Lavrentiev Ave., 8, 630090 Novosibirsk, Russia; malbakhova.inna@yandex.ru (I.Z.); ushakova@1bio.ru (T.U.); gulyaeva00ks@gmail.com (O.G.); morozova@1bio.ru (V.M.); ulona79@mail.ru (Y.K.); tikunova@1bio.ru (N.T.); 2 Faculty of Natural Sciences, Novosibirsk National Research State University, Pirogova St. 2, 630090 Novosibirsk, Russia

**Keywords:** *Pseudomonas aeruginosa*, hydrogel, drug delivery, bacteriophage PA57, human serum albumin, infected wounds

## Abstract

The global proliferation of multidrug-resistant *Pseudomonas aeruginosa* stimulates the search for alternatives to conventional therapy. This study developed human serum albumin (HSA)-based hydrogels for the delivery of bacteriophage PA57. Matrices were fabricated via combined thermal- and ethanol-induced gelation. The release kinetic was dependent on protein concentration: 20% (*w*/*v*) HSA provided sustained release over 48 h, whereas 10–15% (*w*/*v*) HSA exhibited burst release effects. Combined systems effectively suppressed *P. aeruginosa* growth in vitro during the early and middle stages of incubation, maintaining low culture optical density for up to 28 h. Although late-stage bacterial regrowth was observed, the final bacterial load remained significantly lower than in the control. Furthermore, cytocompatibility assays with HaCaT keratinocytes and MRC-5 fibroblasts demonstrated high cell viability, confirming the safety of the hydrogel matrix for wound healing applications. These results demonstrate the promise of HSA-based hydrogels as a platform for localized phage therapy of infected wounds.

## 1. Introduction

The global proliferation of multidrug-resistant bacterial strains critically compromises the efficacy of therapeutic interventions for infected wounds, thereby increasing the risk of systemic complications and placing a significant strain on healthcare systems [1]. Conventional antibiotics and antiseptics are increasingly losing their effectiveness due to rapid pathogen adaptation, limited selectivity of action, and systematic adverse effects [2]. In this context, bacteriophages are considered a promising therapeutic alternative as their lytic activity remains unaffected by bacterial resistance mechanisms, while their high species specificity ensures safety, preservation of the commensal microbiota, and the theoretical potential for synergistic combination with antibiotics, including in immunocompromised patients [3].

Nevertheless, the clinical translation of phage therapy remains constrained by challenges associated with the stabilization and targeted delivery of virions to the wound environment [4]. The integration of bacteriophages into hydrogel matrices has been recognized as one of the most promising strategies to overcome key limitations of phage therapy, including virion instability, rapid washout from the wound bed, and insufficient penetration into bacterial biofilms [5]. In recent years, a number of studies have demonstrated the efficacy of hydrogel-based systems for bacteriophage delivery. Among natural polymers, polysaccharide-based matrices have been the most extensively investigated; for instance, alginate hydrogels fabricated via 3D printing enabled controlled release of phages active against *Escherichia coli*, *Salmonella enterica*, and *P. aeruginosa* over a 24 h period while preserving 81–99% of lytic activity [6,7]. Chitosan-based sponges loaded with anti-*E. coli* phages exhibited bactericidal efficacy, lysing bacterial cultures within 70 ± 10 min, with lyophilized formulations retaining activity for up to 12 weeks [8]. However, polysaccharide matrices possess inherent limitations: a dense polymer network may impede the diffusion of large phage particles, while electrostatic interactions with charged polymer functional groups can potentially lead to virion inactivation [9,10]. However, polysaccharide matrices possess inherent limitations when applied to phage delivery. The dense polymer network of ionotropically crosslinked alginate (pore size typically below 100 nm) [11] may physically impede the diffusion of large phage virions, resulting in incomplete release and reduced bioavailability at the infection site. Furthermore, the preparation of chitosan-based matrices typically requires acidic conditions (pH 4.0–5.5) since chitosan is only soluble in dilute aqueous acids such as acetic acid. Such an acidic environment can be detrimental to phages, promoting overall loss of infectivity. In addition, conventional gelation strategies often involve aggressive chemical cross-linkers (e.g., glutaraldehyde, genipin) or harsh physical treatments, which may further compromise virion integrity and reduce the biological activity of the encapsulated phages.

Protein hydrogels exhibit high biocompatibility, biodegradability, and the ability to closely mimic the native extracellular matrix, thereby creating a favorable microenvironment for tissue regeneration [12,13,14]. For bacteriophage immobilization, protein-based matrices are particularly advantageous due to their abundance of functional groups for controlled virion loading, capacity for reversible conformational rearrangement during gelation, and potential for enzyme-triggered release within the wound environment. These characteristics minimize the risk of phage inactivation and enable sustained therapeutic efficacy [15,16,17]. For example, gelatin hydrogel microspheres have achieved nearly 100% loading efficiency for phage T4, releasing approximately 75% of the encapsulated particles within 6 h, and have been successfully utilized for the co-delivery of phages and basic fibroblast growth factor (bFGF) to promote wound healing [16]. Similarly, gelatin–alginate composite hydrogels incorporating anti-P. aeruginosa phages have demonstrated superior virion adsorption and release kinetics compared with pure alginate matrices [17]. Tao et al. developed an injectable hydrogel comprising gelatin crosslinked with polyethylene glycol. A defining feature of this system is its structural stability in healthy tissues, coupled with rapid degradation upon exposure to P. aeruginosa-secreted gelatinases, which facilitates the targeted delivery of a phage cocktail (FJK.R9-30 and MK.R3-15) directly to the infection site [18].

Among protein-based carriers, human serum albumin (HSA) offers a unique combination of structural, transport, and protective properties that distinguish it fundamentally from gelatin, collagen, and polysaccharide matrices. First, HSA possesses multiple ligand-binding sites [19,20]. Third, the free thiol group of Cys34 confers intrinsic antioxidant capacity to HSA, potentially shielding encapsulated phages from oxidative stress within the wound microenvironment [21]. Fourth, HSA is the most abundant plasma protein (60% of total serum protein), ensuring exceptional biocompatibility and biodegradability under physiological conditions [22]. Previously, we developed a methodology for fabricating HSA-based hydrogels via combined thermal- and ethanol-induced gelation in a phosphate-buffered solution. This approach preserved the functional sites of the protein; enabled a broad range of tunable rheological properties; and demonstrated high cytocompatibility, structural stability, and antibacterial efficacy upon loading with low molecular weight antibiotics [23]. In the present study, we investigate for the first time the incorporation of the lytic bacteriophage PA57 into HSA hydrogels fabricated using this combined thermal- and ethanol-induced method. We hypothesize that the reversible conformational rearrangement of HSA will preserve virion infectivity, while the density of the resulting protein network will modulate the release kinetics of the phage. To test this hypothesis, the following objectives were established: (1) to evaluate the stability of the phage titer following encapsulation in hydrogels with varying HSA concentrations, (2) to construct release kinetics profiles, and (3) to assess the efficiency of *P. aeruginosa* eradication in a simulated wound environment. The findings of this study will provide a foundation for the rational design of HSA-based, phage-loaded hydrogels intended for the therapy of infected wounds.

## 2. Results and Discussion

### 2.1. Assessment of Lytic Activity and Morphological Characteristics of Bacteriophage PA57

This section presents the results of a comprehensive phenotypic and morphological characterization of bacteriophage PA57, along with an in vitro evaluation of its lytic kinetics. To determine the lytic activity spectrum of bacteriophage PA57, its efficacy was evaluated against a panel of 75 *P. aeruginosa* isolates. Complete lysis of the bacterial lawn (high lytic activity) was observed for 23 strains (30.7%), whereas 13 isolates (17.3%) exhibited the formation of single plaques or partial clearing of the medium (weak activity). No lytic activity was detected in 39 cases (52.0%). These results indicate that phage PA57 possesses a pronounced lytic potential against a significant proportion of *P. aeruginosa* populations, highlighting its promise as a therapeutic agent for the development of phage therapy preparations. The reference strain *P. aeruginosa* CEMTC 670 was selected for in-depth biological, morphological, and kinetic characterization as it demonstrates high susceptibility to PA57 and is of greatest clinical interest in the context of combating highly resistant infections. The obtained data provide insights into the phage’s host range, structural features, and lytic efficacy against clinically relevant *P. aeruginosa* strains, representing a critical prerequisite for justifying its potential application in phage therapy.

**Figure 1:**
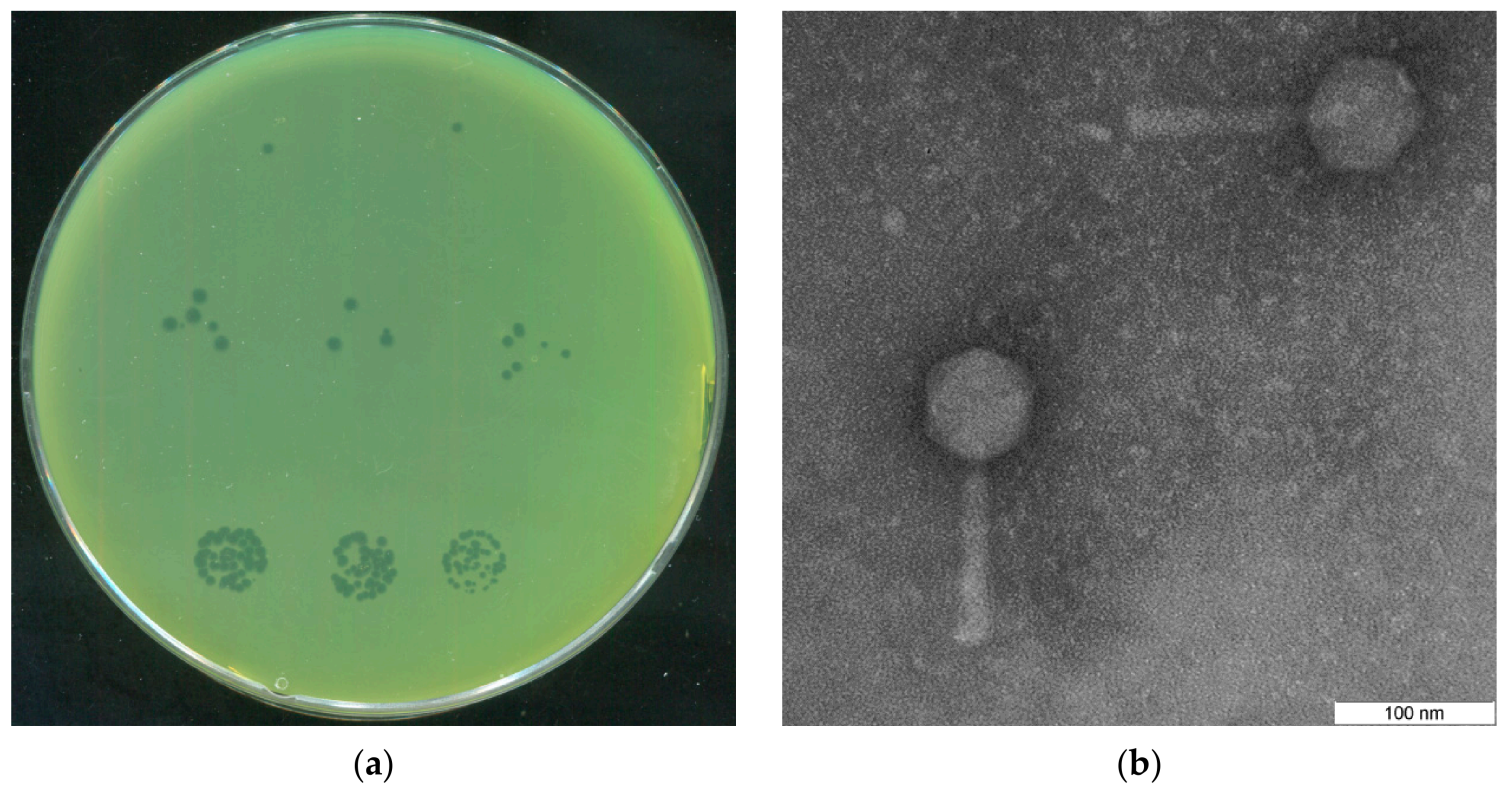
Determination of the lytic activity of bacteriophage PA57 against *P. aeruginosa* strain CEMTC 670 and its morphology: (**a**) Transparent lysis zones formed on the surface of agarized nutrient medium following spot application of tenfold serial dilutions of phage lysate with titers of 10^2^, 10^3^, and 10^4^ PFU/mL (arranged top to bottom). (**b**) Transmission electron micrographs of phage PA57 virions.

For quantitative assessment of lysis zone morphology, tenfold serial dilutions of the phage lysate (10^2^, 10^3^, and 10^4^ PFU/mL) were spotted onto an agarized medium seeded with a bacterial lawn of *P. aeruginosa* strain CEMTC 670. Incubation revealed the formation of distinct, transparent plaques of small diameter across all tested dilutions, confirming stable lytic activity of the phage against this isolate (Figure 1). Quantitative analysis demonstrated a mean plaque diameter of 0.124 ± 0.02 cm (n = 20). The low standard deviation indicates homogeneity of the phage particle population and reproducibility of lysis zone formation. The transparent plaque morphology confirms that phage PA57 follows a strictly lytic developmental cycle, which represents a critical criterion for potential therapeutic applications.

**Figure 2:**
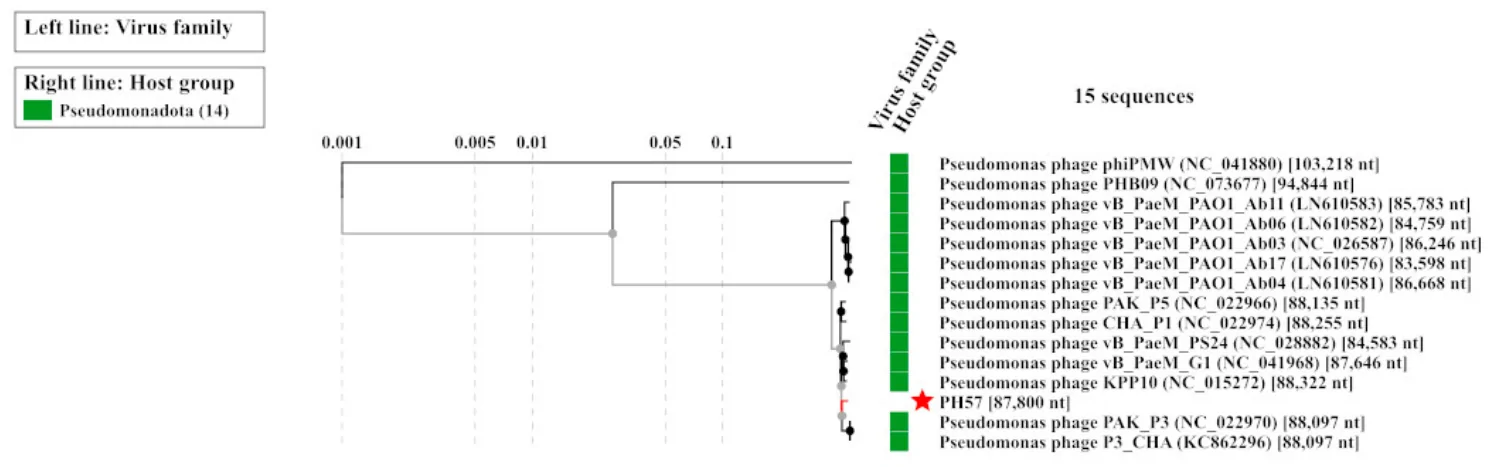
Phylogenetic tree generated using ViPTree based on whole-proteome similarity between phage PA57 (marked with a red star) and related *Pseudomonas* phages.

Phage PA57 genome length was determined as 87,800 bp; virulence factors and antibiotic resistance genes were not found in this genome using search in the Virulence Factor (VR) and Antibiotic Resistance Gene (AGR) databases, respectively. BACPHLIP software predicted the lytic lifestyle for the phage PA57 (Figure 2). The ViPTree analysis suggests that phage belongs to the *Vandenendeviridae* family, *Nankokuvirus* genus. Phages from this genus were previously described as virulent [24].

**Figure 3:**
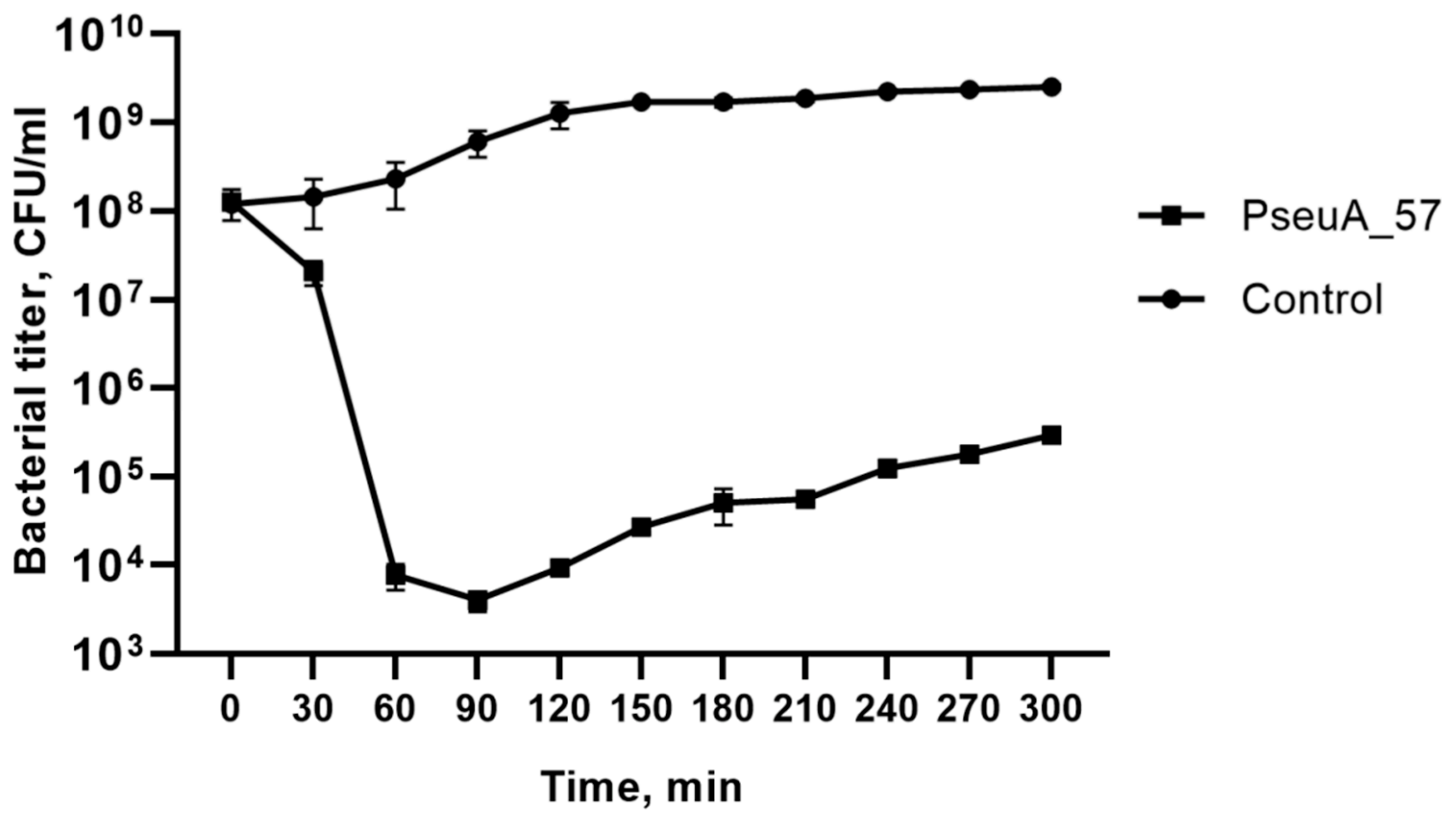
Lytic kinetics of bacteriophage PA57 against a suspension culture of *P. aeruginosa* CEMTC 670. The experiment was performed at an MOI = 0.1. Squares: experimental group (inoculated with phage PA57); circles: control group (no phage added).

The lytic kinetics of bacteriophage PA57 against a liquid culture of *P. aeruginosa* CEMTC 670 were evaluated at a multiplicity of infection (MOI) of 0.1. Upon addition of phage particles, a rapid decline in viable cell count was observed: within 90 min, the colony-forming unit (CFU) concentration decreased by five orders of magnitude, from 1.2 × 10^8^ to 3.8 × 10^3^ CFU/mL (Figure 3). The minimum bacterial titer was recorded at 90 min post-infection, followed by a gradual increase in bacterial titer. This regrowth may be attributed to the emergence of phage-resistant variants, gradual depletion of infectious phage particles, or other adaptive mechanisms; however, the specific cause was not experimentally confirmed in the current study (e.g., by isolating regrown colonies and testing their phage susceptibility).

In summary, bacteriophage PA57 exhibits potent and rapid lytic activity against *P. aeruginosa* CEMTC 670, as evidenced by the formation of homogeneous, transparent plaques and a ≥5-log reduction in viable cell count within 90 min in vitro. The strictly lytic lifestyle, confirmed by plaque morphology and transmission electron microscopy, fulfills a critical safety criterion for therapeutic candidates.

### 2.2. Development of HSA-Based Hydrogels Loaded with Bacteriophage PA57

Building upon our previous work on the fabrication of HSA-based hydrogels [23], this study presents the development of an antimicrobial platform for the treatment of infected wounds associated with multidrug-resistant pathogens. In the present work, the lytic bacteriophage PA57, specific against *P. aeruginosa*, was incorporated into the HSA hydrogel matrix. The aim of this study was to evaluate the effect of phage loading on the structural and mechanical properties of the hydrogel, the kinetics of viral particle release, and the efficacy of *P. aeruginosa* cell eradication in vitro.

**Figure 4:**
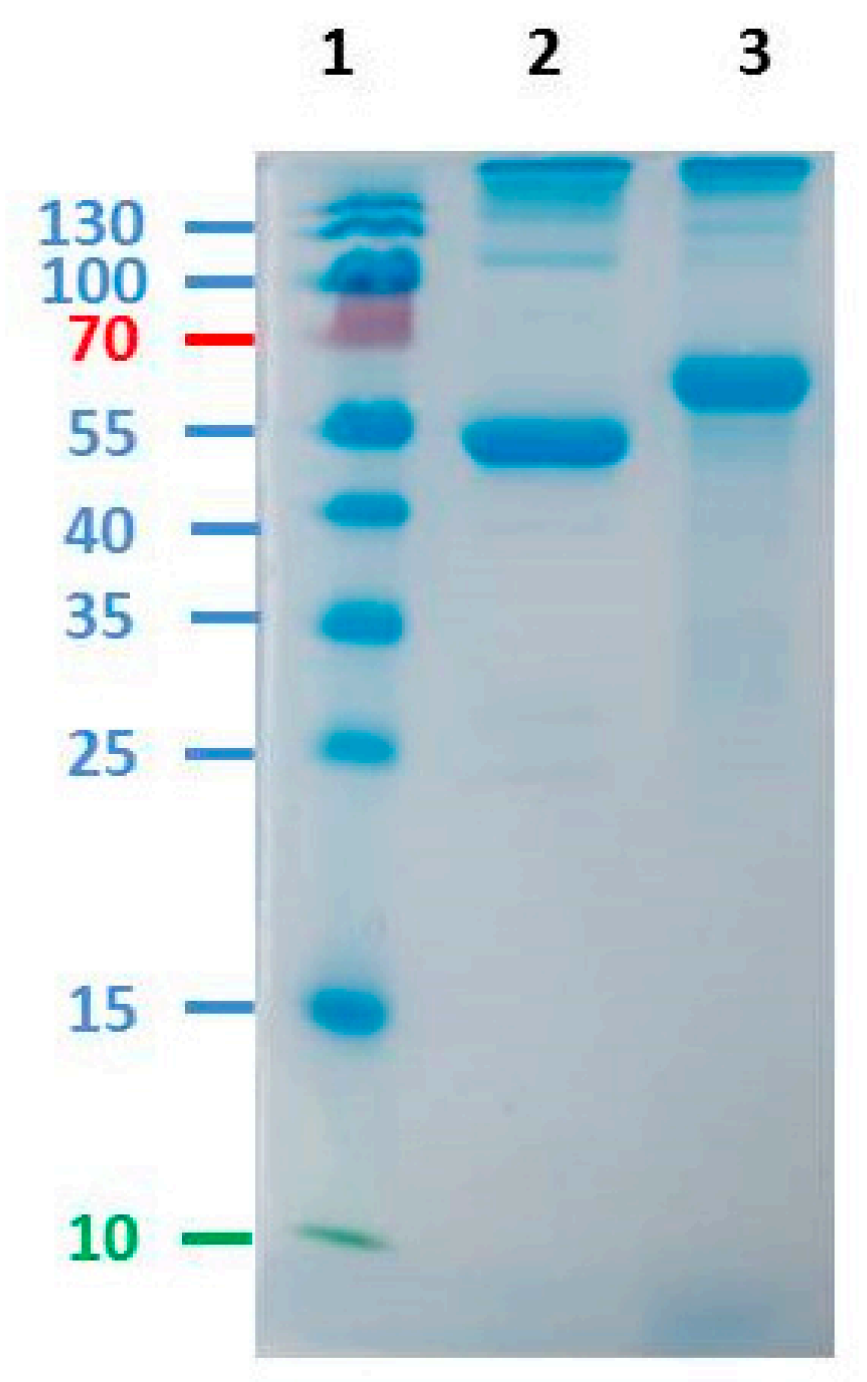
SDS-PAGE analysis of HSA under reducing and non-reducing conditions. Lane 1: molecular weight markers; lane 2: HSA (2 μg) without β-mercaptoethanol treatment (non-reducing conditions); lane 3: HSA (2 μg) treated with β-mercaptoethanol (reducing conditions).

To ensure the quality of the starting HSA material, SDS-PAGE analysis was performed under both reducing and non-reducing conditions (Figure 4). Under both conditions, the monomeric form (~65 kDa and ~55 kDa, respectively) predominated, accounting for ≥80% of the total protein content. The observed shift in electrophoretic mobility is attributed to conformational changes associated with the partial reduction of 17 intramolecular disulfide bonds [25]. The absence of low-molecular-weight fragments confirms the structural integrity of the protein preparation and its suitability for hydrogel matrix fabrication.

**Figure 5:**
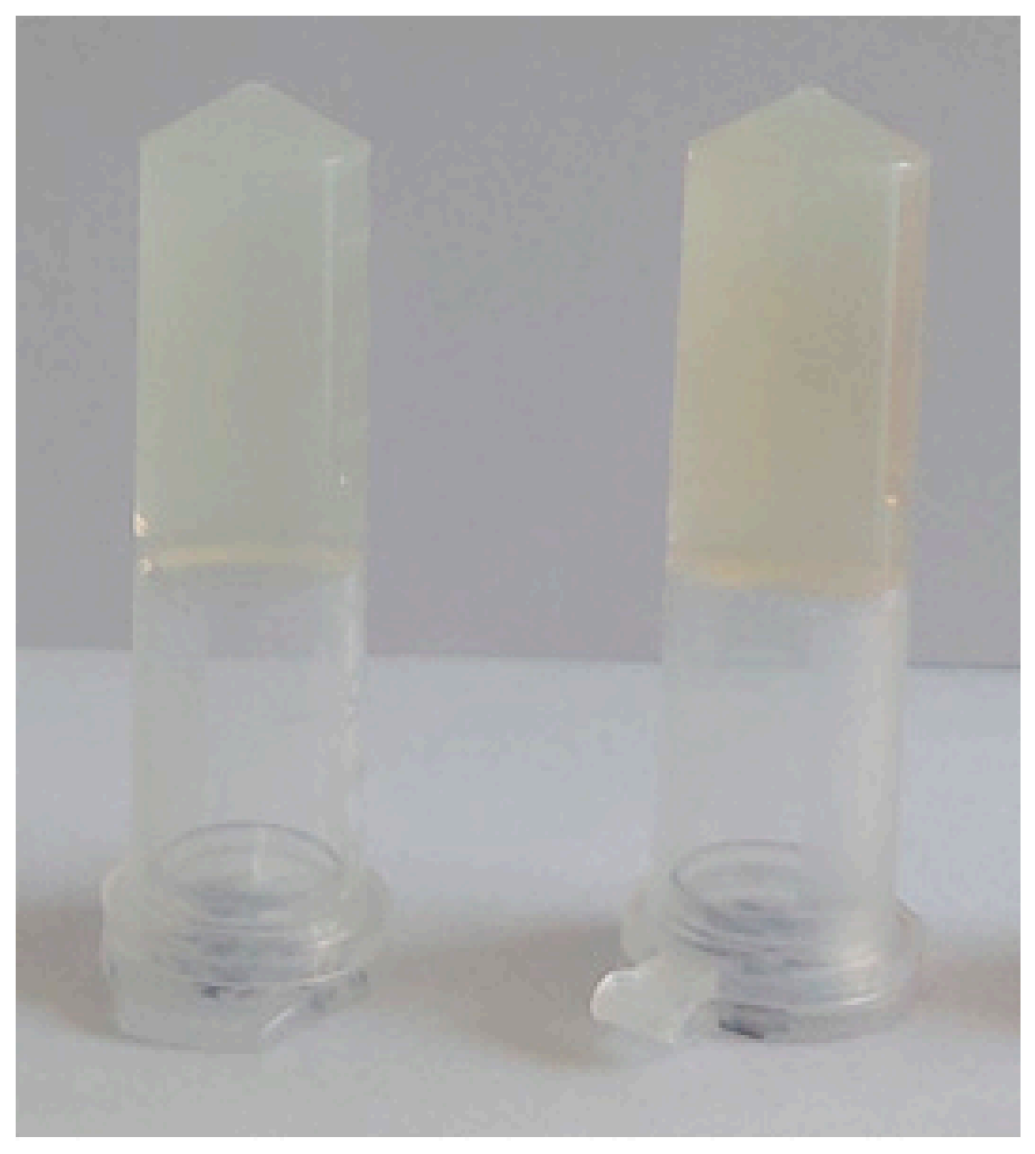
Macroscopic appearance of HSA-based hydrogels. Samples are arranged from left to right: (1) control hydrogel composed of 15% (*w*/*v*) HSA and 15% (*v*/*v*) ethanol; (2) hydrogel loaded with bacteriophage PA57 (15% (*w*/*v*) HSA, 10% (*v*/*v*) ethanol, 10^8^ PFU/mL). No visible differences in transparency, color, or structural integrity were observed between formulations.

Visual inspection of the prepared hydrogels (Figure 5) confirmed the preservation of structural integrity and homogeneity across all experimental groups. The control sample (far left), comprising a matrix of 15% (*w*/*v*) HSA and 15% (*v*/*v*) ethanol, formed a stable, transparent gel with a slight yellowish-white hue. Upon incorporation of bacteriophage PA57 at a concentration of 10^8^ PFU/mL (central sample), no alterations in macroscopic characteristics were observed: the gel retained its shape, transparency, and original coloration. These findings indicate full compatibility between the viral suspension and the protein-based matrix, with no evidence of visible particle aggregation.

**Figure 6:**
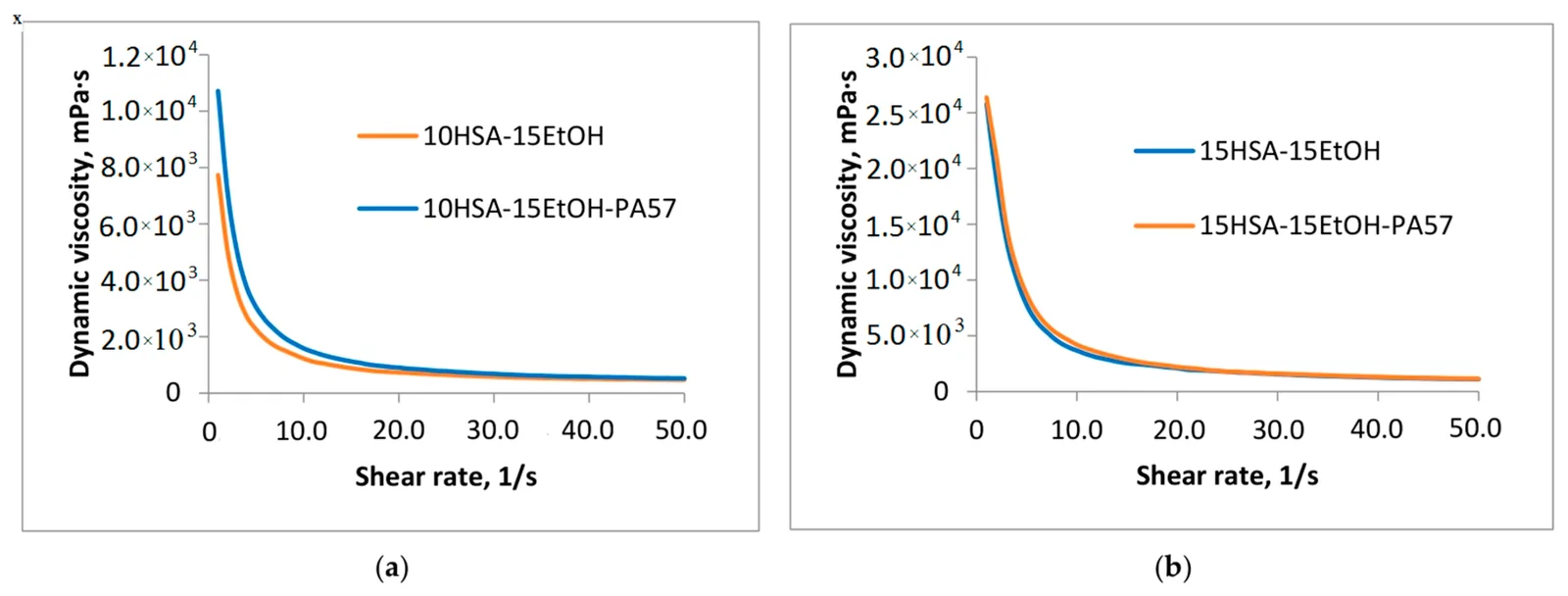
Dependence of dynamic viscosity on shear rate for human serum albumin (HSA)–ethanol-based hydrogels. (**a**) 10HSA-15EtOH formulation. (**b**) 15HSA-15EtOH formulation. Graphs display control samples (unloaded matrices) and hydrogels loaded with bacteriophage PA57 (10^8^ PFU/mL). Color coding of curves: in panel (**a**), red indicates control, and blue indicates PA57; in panel (**b**), blue indicates control, and red indicates PA57. Both systems exhibit pronounced shear-thinning (pseudoplastic) behavior, characterized by a marked decrease in viscosity with increasing shear rate and plateau formation at shear rate > 20 s^−1^. Incorporation of phage particles does not induce significant alterations in the rheological profile, confirming the structural compatibility of the viral suspension with the protein–ethanol matrix.

The flow behavior of the prepared hydrogels was evaluated by measuring dynamic viscosity as a function of shear rate (Figure 6). Both formulations exhibited pronounced shear-thinning (pseudoplastic) behavior, characterized by a steep decline in viscosity at low shear rates (0–10 s^−1^) followed by a gradual plateau at higher shear rates (up to 50 s^−1^). This non-Newtonian flow profile is typical of structured protein-based networks and is highly advantageous for topical wound applications as it facilitates easy spreading under mechanical stress while preserving structural integrity at rest. Baseline viscosity was strongly dependent on the HSA concentration. The 15HSA-15EtOH (15% (*w*/*v*) HSA and 15% (*v*/*v*) ethanol) formulation demonstrated significantly higher initial viscosity compared with the 10HSA-15EtOH (10% (*w*/*v*) HSA and 15% (*v*/*v*) ethanol) system, reflecting increased polymer density and enhanced physical entanglement within the matrix. Incorporation of bacteriophage PA57 (10^8^ PFU/mL) exerted a formulation-dependent effect on rheological properties. In the 10HSA-15EtOH hydrogel, phage loading resulted in a modest but consistent elevation in viscosity across the entire shear rate range, suggesting potential weak electrostatic or steric interactions between viral capsids and the albumin network that slightly reinforce gel cohesion. Conversely, in the denser 15HSA-15EtOH formulation, the viscosity profiles of the unloaded control and PA57-loaded hydrogel were nearly superimposable, indicating that the higher protein concentration dominates the macroscopic flow response and effectively masks any minor structural perturbations induced by phage incorporation. Overall, the preserved shear-thinning behavior and minimal viscosity alteration upon viral loading confirm that PA57 integration does not compromise the fundamental rheological performance of the platform, supporting its suitability for controlled topical delivery and wound coverage.

**Figure 7:**
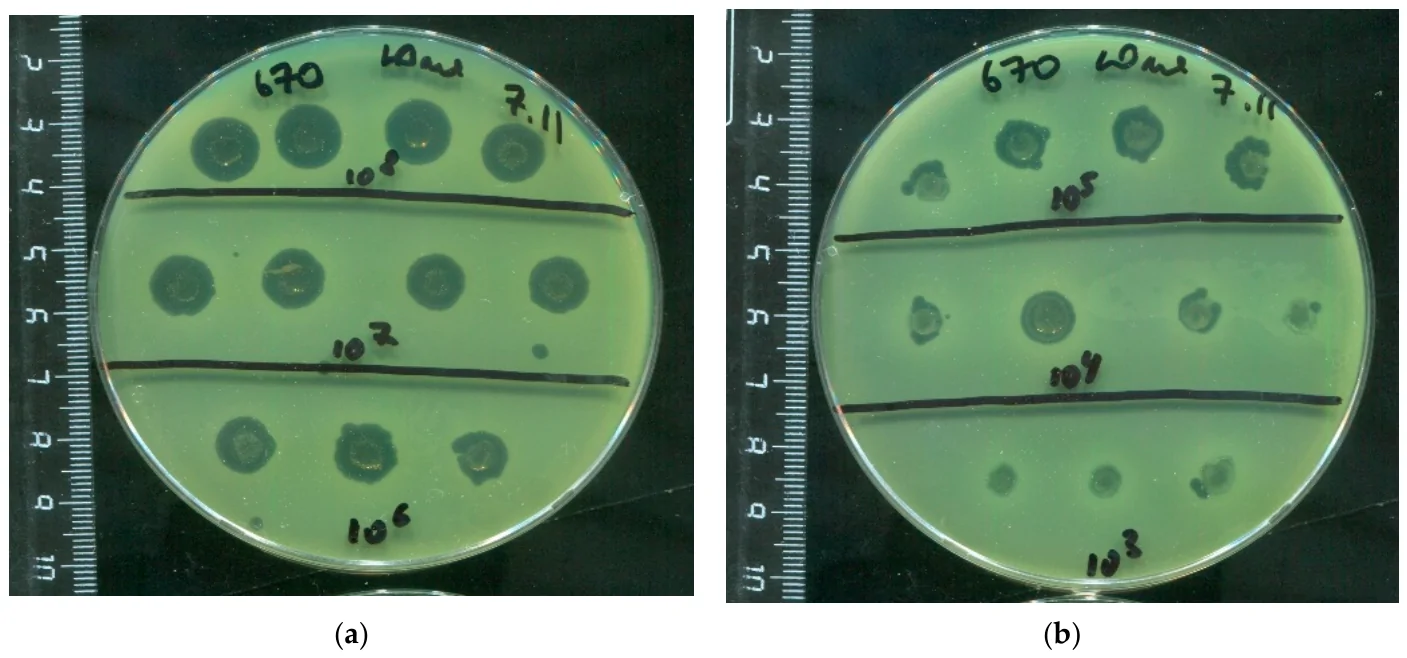
Lysis zones formed by bacteriophage PA57 released from HSA-based hydrogels. Hydrogels loaded with the indicated phage titers were spotted onto *P. aeruginosa* CEMTC 670 bacterial lawns and incubated for 24 h at 37 °C. Panels show zones of complete lysis (≥10^6^ PFU/mL) (**a**) and partial/absent lysis (<10^6^ PFU/mL) (**b**). Scale bar: 1 cm.

To assess the functional integrity of bacteriophage PA57 following incorporation into the HSA-based hydrogel matrix, a spot lysis assay was performed against *P. aeruginosa* strain CEMTC 670 (Figure 7). Hydrogels (15% (*w*/*v*) HSA and 15% (*v*/*v*) ethanol) were loaded with serial dilutions of PA57 (10^3^–10^8^ PFU/mL), spotted onto bacterial lawns, and incubated for 24 h at 37 °C. As shown in the Figure 7 and Table 1, hydrogels containing ≥10^6^ PFU/mL produced well-defined zones of complete bacterial lysis with smooth margins, indicating efficient diffusion of infectious virions from the protein matrix and preservation of receptor-binding capacity in the released particles. At intermediate titers (10^4^–10^5^ PFU/mL), lysis zones were smaller and exhibited irregular boundaries, consistent with sub-saturating phage concentrations and localized diffusion constraints. No lysis was detected at the lowest loading (10^3^ PFU/mL), establishing the functional detection threshold under these experimental conditions.

**Figure 8:**
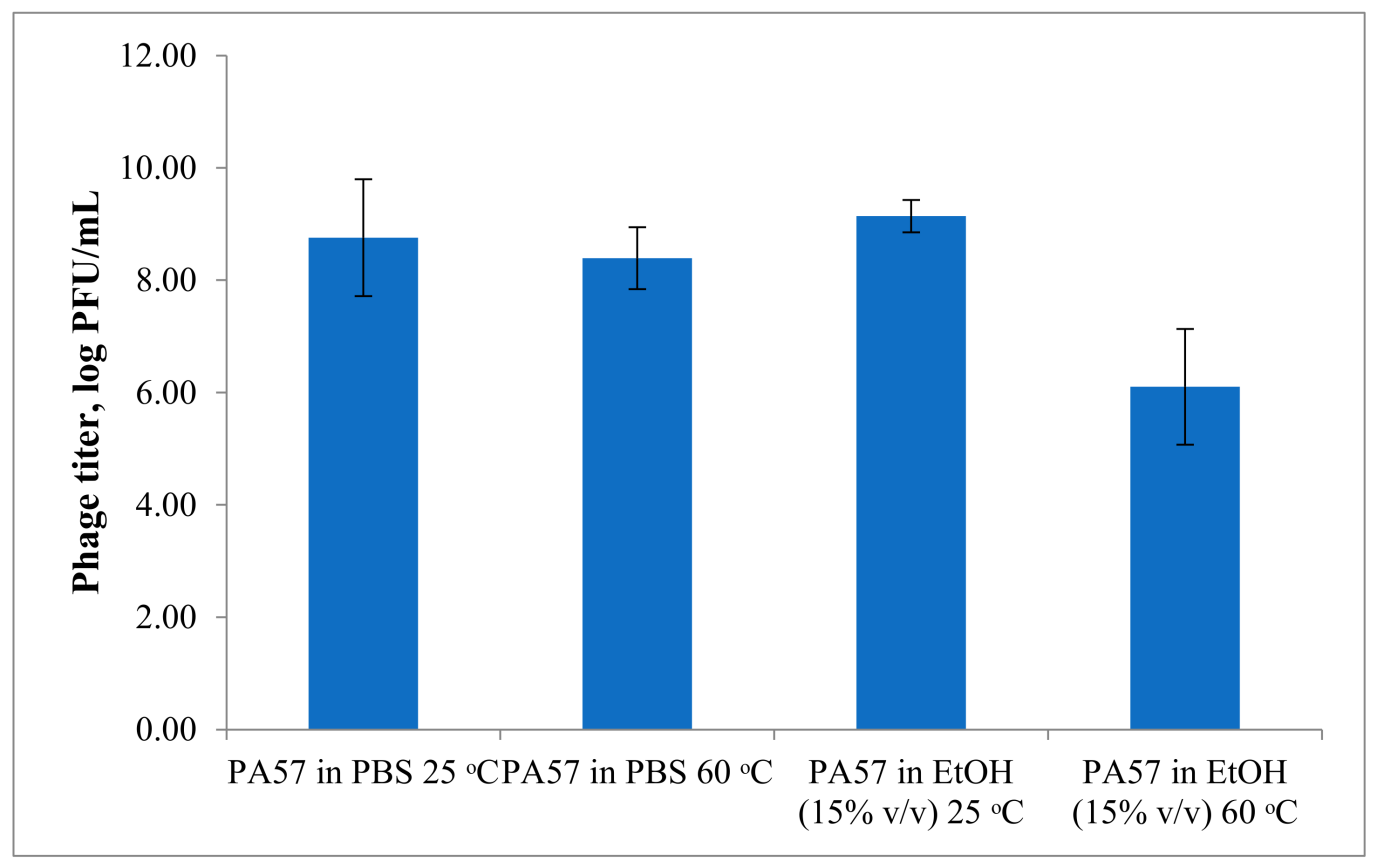
Stability of bacteriophage PA57 under various experimental conditions. The initial phage titer was 5 × 10^8^ PFU/mL. Incubation conditions: (1) PBS, 25 °C (control); (2) PBS, 60 °C, 15 min; (3) PBS + 15% (*v*/*v*) EtOH, 25 °C; (4) PBS + 15% (*v*/*v*) EtOH, 60 °C, 15 min.

Stability assessment of bacteriophage PA57 under hydrogel formation conditions was performed using an initial phage titer of 5 × 10^8^ PFU/mL. As shown in Figure 8, under control conditions (PBS, 25 °C), the phage retained high infectious activity (~8.8 log PFU/mL). Exposure to elevated temperature (60 °C) in the absence of organic solvents resulted in only a marginal titer reduction (~8.4 log), indicating adequate thermostability of the phage particles. Similarly, the presence of 15% ethanol at room temperature exhibited no inhibitory effect; moreover, the titer under these conditions was comparable to or even slightly exceeded the control value (~9.2 log PFU/mL). However, the combined exposure to heat (60 °C) and ethanol (15% *v*/*v*) caused a substantial decline in infectious activity, with the titer dropping to ~6.1 log PFU/mL. These findings demonstrate that bacteriophage PA57 is resistant to individual stress factors but exhibits sensitivity to their synergistic action.

Given the observed sensitivity of the phage to the combined action of temperature and ethanol, we further evaluated the capacity of the HSA-based hydrogel matrix to protect viral particles under conditions mimicking their incorporation and subsequent release. To simulate complete phage release from the hydrogel, the samples were resuspended by vortexing in a buffer, followed by the determination of the phage titer in the resulting solution. It was found that at an initial phage loading of 2 × 10^8^ PFU/mL, the highest recovery of infectious particles was observed for the formulation with the highest protein concentration (20HSA-15EtOH): 10.3 ± 2.2% of the initial titer. For the 15HSA-15EtOH formulation, the recovery was 3.3 ± 1.5%, whereas for the hydrogel with the lowest albumin content (10HSA-15EtOH), it amounted to only 1.3 ± 0.6% (Table 2). Notably, the 10HSA-15EtOH and 15HSA-15EtOH samples exhibited relatively high variability in phage titer retention. Thus, increasing the HSA concentration in the hydrogel formulation is associated with enhanced preservation and subsequent release of viable phage particles. It should be emphasized that this concentration-dependent effect establishes a correlation rather than indicating a specific causal mechanism. We hypothesize that a denser protein network may create a more favorable local microenvironment that limits virion exposure to inactivating factors during gelation. However, alternative explanations for the observed results cannot be excluded (e.g., differences in the efficiency of phage extraction from the matrix or a specific protective effect of HSA on phage virions), and the exact underlying mechanism was not directly elucidated in the present study.

**Figure 9:**
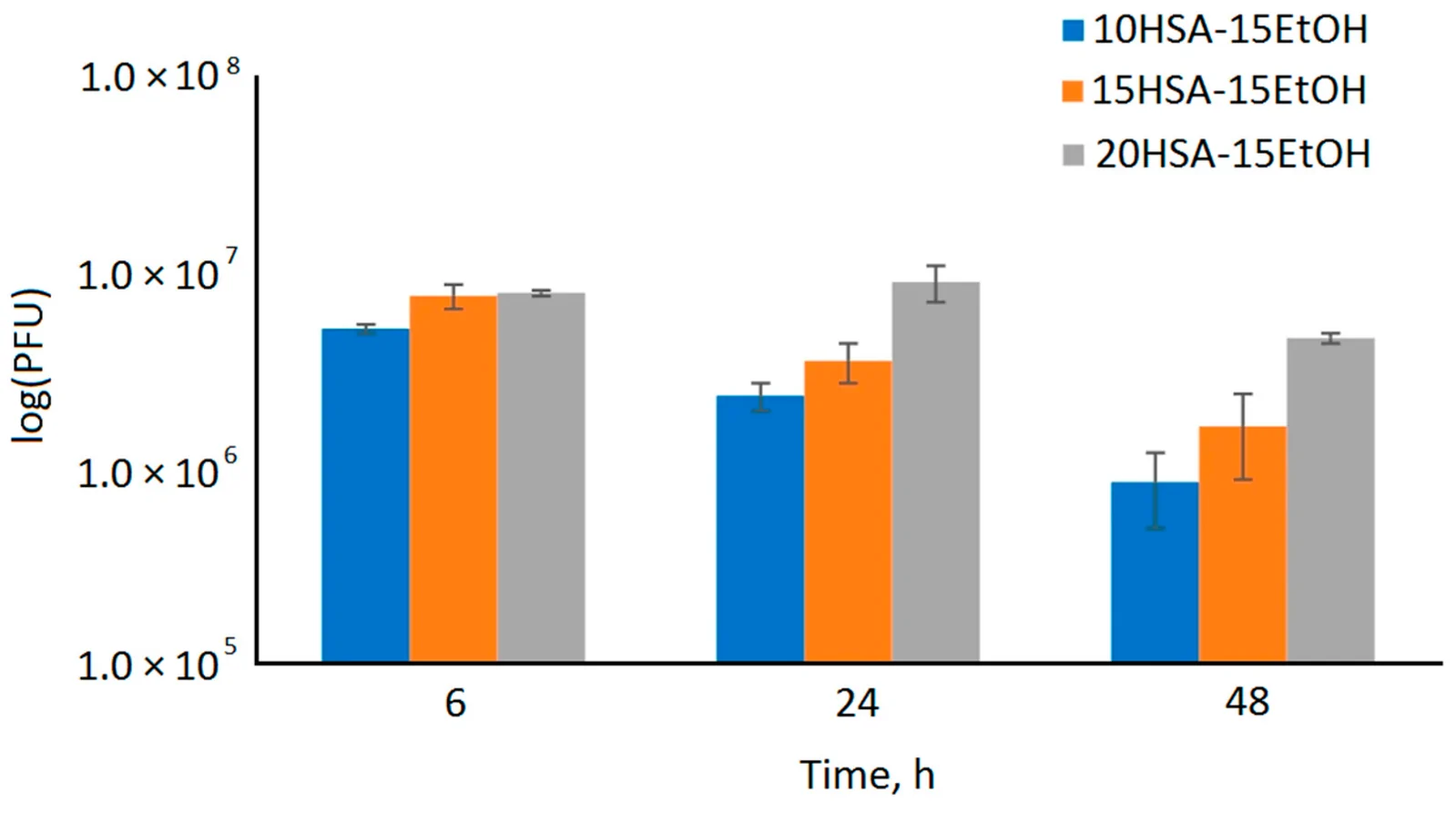
Time-dependent release of bacteriophage PA57 from HSA-based hydrogels.

Kinetic analysis of phage release was performed under dynamic sink conditions to simulate the physiological wound environment (Figure 9). It is important to note that Figure 8 presents non-cumulative release data (i.e., the phage concentration measured at each individual time point), rather than cumulative release data (where the total amount of phage released up to each time point is summed). The release profiles demonstrated distinct patterns depending on the density of the polymer network. Hydrogels with lower HSA concentrations (10% and 15% *w*/*v*) exhibited a pronounced initial burst release within the first 6 h, followed by a gradual decline in the release rate at 24 and 48 h. In contrast, the denser 20HSA-15EtOH formulation demonstrated a sustained release profile, characterized by a less pronounced initial burst and a delayed release peak observed at 24 h (9.08 × 10^6^ PFU in the sampled aliquot). Over the 48 h period, the cumulative release relative to the initially loaded phage titer (without accounting for the loss of phage activity during hydrogel formation) amounted to 2.1%, 3.2%, and 5.4% for the 10HSA, 15HSA, and 20HSA formulations, respectively. Taken together, these observations are consistent with a dual protective and modulatory role of the HSA matrix; however, this interpretation should be regarded as a working hypothesis rather than a directly demonstrated mechanism. The higher recovery of infectious phage from denser formulations may indicate that the protein network partially mitigates the synergistic inactivating effects of ethanol and heat during gelation, while the increased density of the 20HSA formulation appears to create a diffusion barrier. This barrier effectively attenuates the initial burst effect and prolongs the elution of infectious phage particles, which is a critical feature for localized therapeutic systems requiring sustained antimicrobial activity at the infection site.

**Figure 10:**
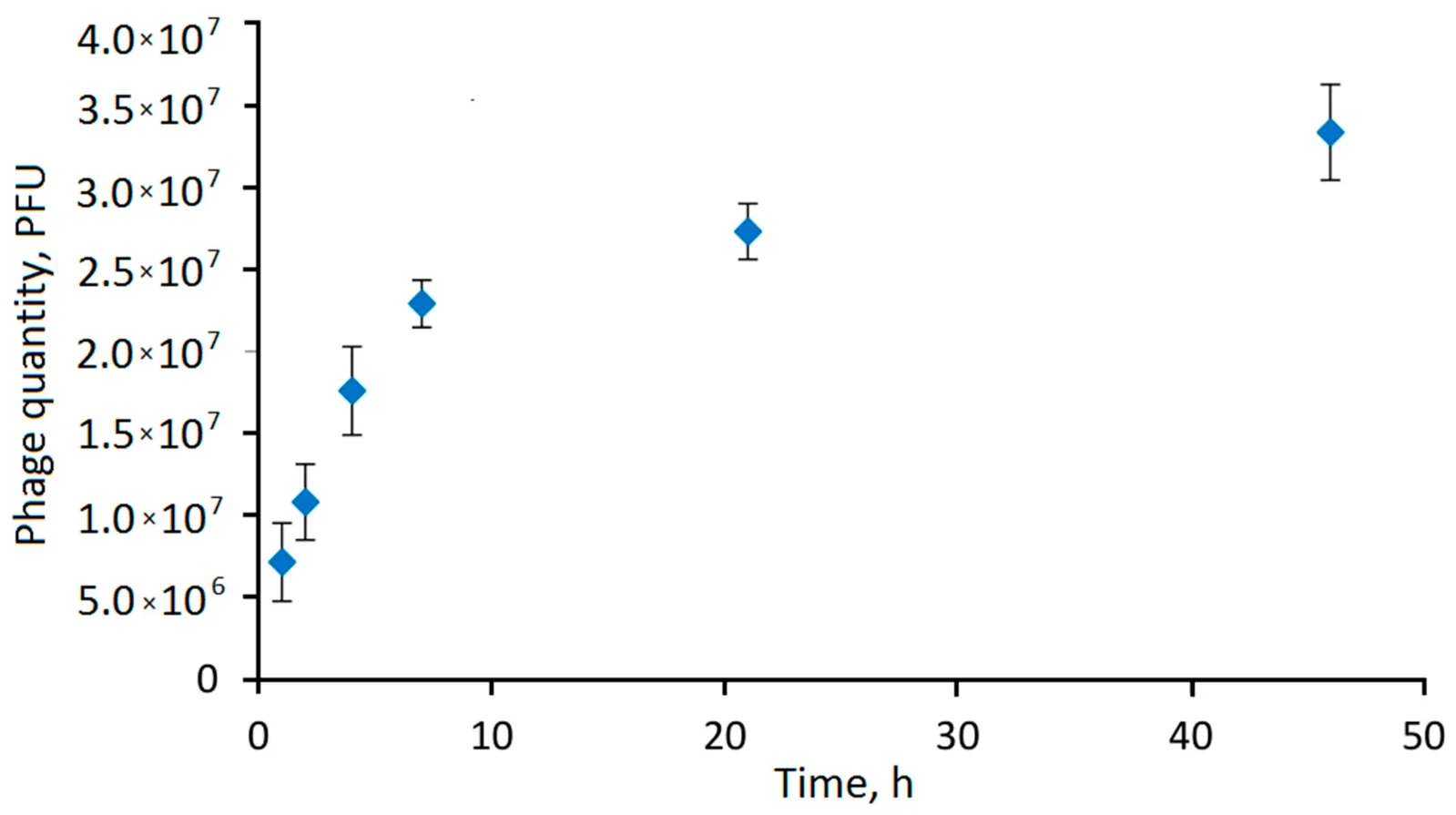
Cumulative release of bacteriophage PA57 from 20HSA-15EtOH hydrogel under dynamic flow conditions (PBS, 37 °C, 300 rpm).

**Figure 11:**
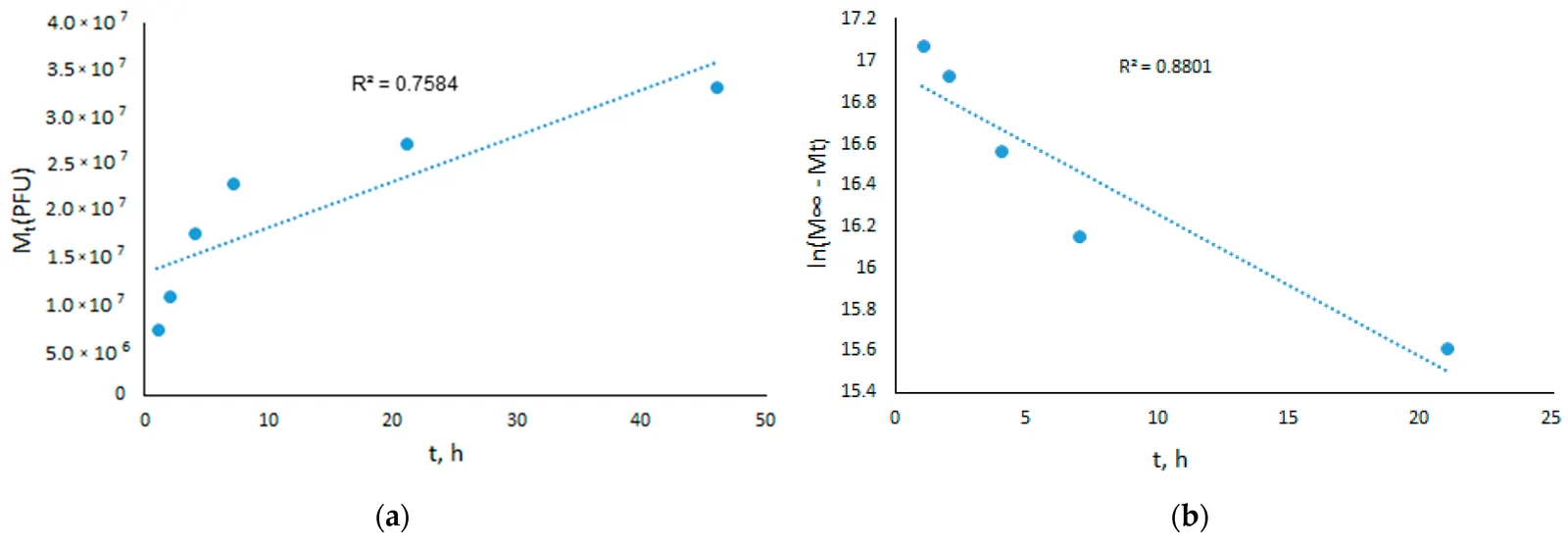

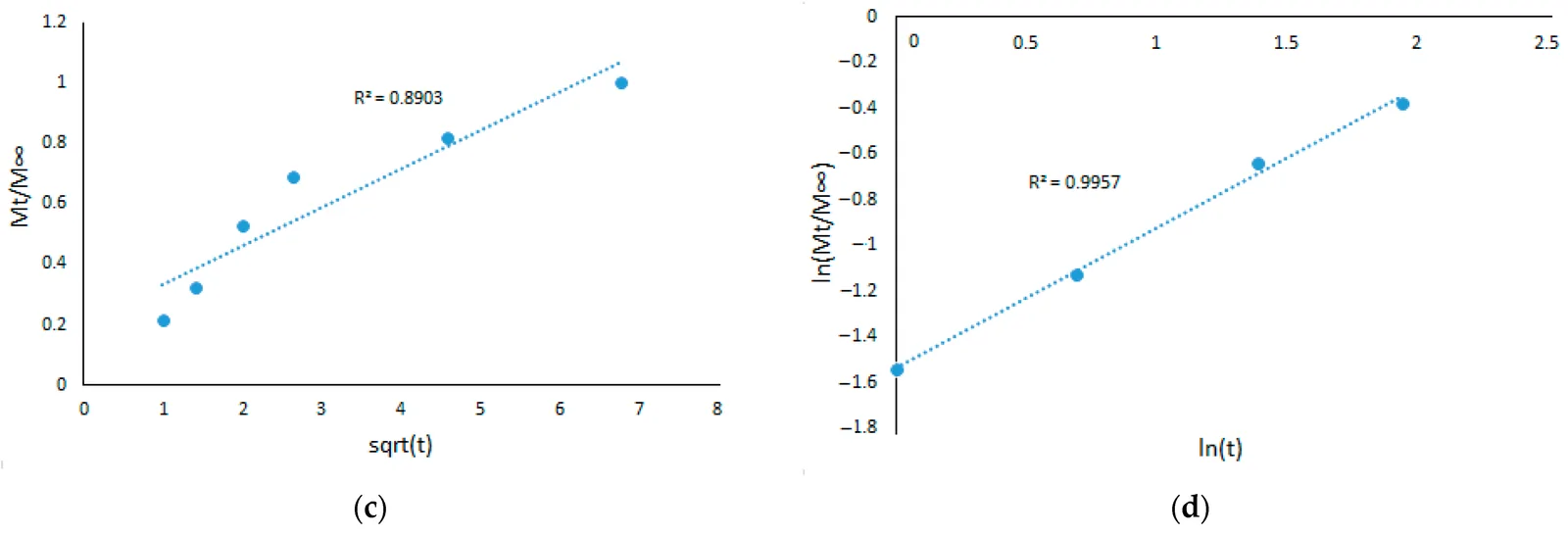
Linearization of cumulative release data for phage PA57 from 20HSA-15EtOH hydrogel matrix: (**a**) zero-order, (**b**) first-order, (**c**) Higuchi, and (**d**) Korsmeyer–Peppas.

To quantitatively describe the release kinetics of phage PA57 from the developed 20HSA-15EtOH hydrogel (Figure 10), a comparative analysis of four kinetic models was performed: zero-order, first-order, Higuchi, and Korsmeyer–Peppas. Release data were analyzed over a time range of 1 to 46 h (Table 3, Figure 11). Notably, following the complete resuspension of the 20HSA-15EtOH hydrogel to recover the remaining phages, the average proportion of phages released during the kinetic elution amounted to 49.7 ± 3.4%. Given that the Korsmeyer–Peppas model is theoretically applicable only to the initial 60% of the release process, the kinetic analysis was performed using data from the 1 to 7 h period, corresponding to Mt/M∞ values ranging from 0.21 to 0.69. To assess the goodness of fit of each model to the experimental data, the coefficient of determination (R^2^) was calculated, and the results are presented in the table. As shown in Table 3, the Korsmeyer–Peppas model provides the best fit to the experimental data for the 20HSA-15EtOH formulation (R^2^ = 0.9957). The release exponent in the Korsmeyer–Peppas model was n = 0.61, which falls within the range characteristic of anomalous transport (0.45 < n < 0.89). This indicates that phage release from the dense 20% (*w*/*v*) HSA matrix is governed by a combined mechanism involving both Fickian diffusion of virions through the protein network and gradual relaxation or partial swelling of the matrix, which is consistent with the prolonged release profile observed over approximately 48 h.

**Figure 12:**
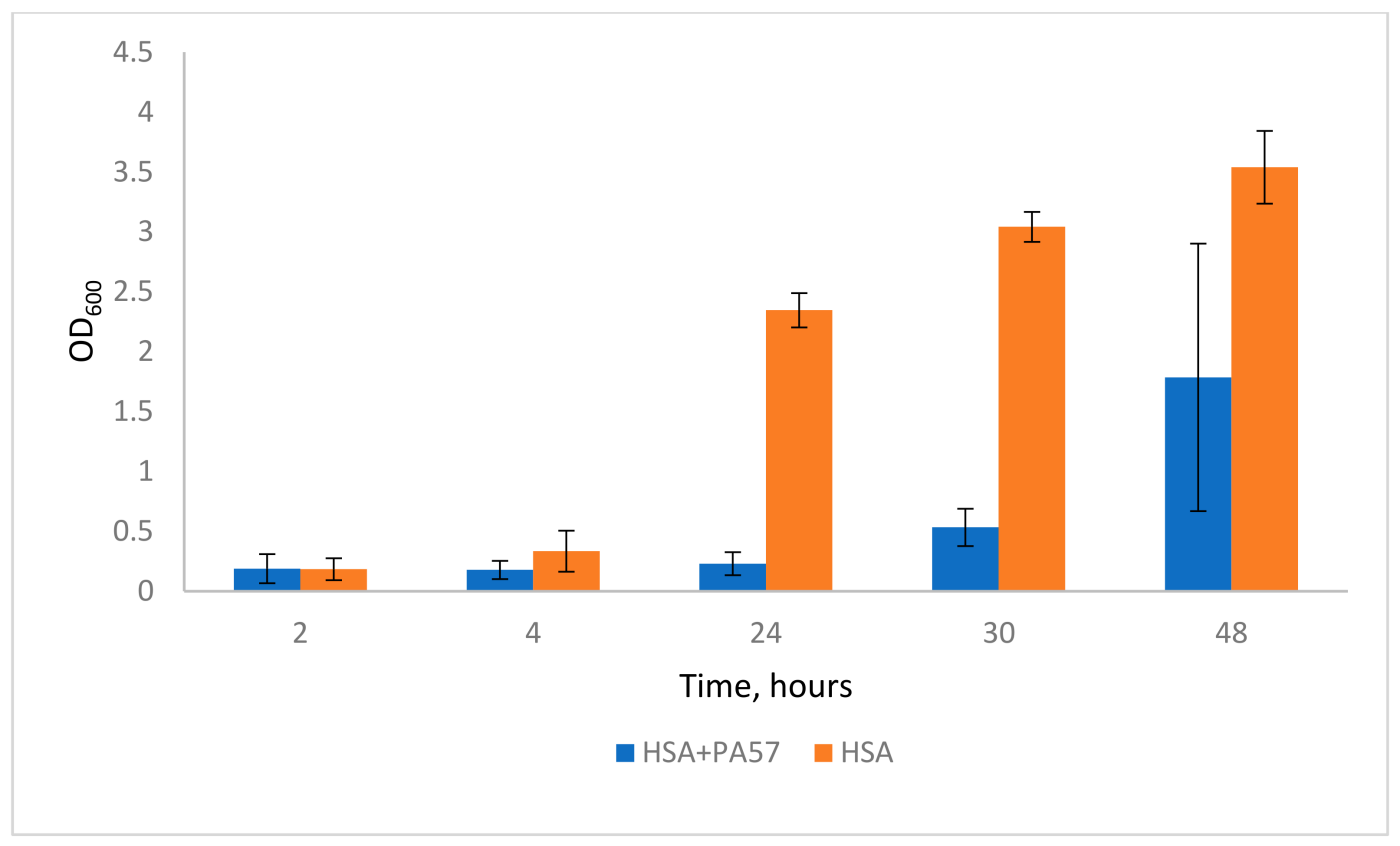
Growth kinetics of planktonic *P. aeruginosa* culture (CEMTC 670) in the presence of HSA-based hydrogels. The optical density at 600 nm (OD_600_) was monitored over time. Series designations: HSA—empty 15% (*w*/*v*) HSA hydrogel (without phage); HSA+PA57—hydrogel 15% (*w*/*v*) HSA loaded with phage PA57 (10^8^ PFU/mL). No significant differences were observed at early time points (2 h: mean difference 0.005, adj. *p* > 0.9999; 4 h: −0.157, adj. *p* = 0.9741), whereas from 24 h onward, bacterial growth suppression in the PA57 group was highly significant (*p* < 0.0001).

To evaluate the efficiency of *P. aeruginosa* elimination in bulk culture, bacterial suspensions were incubated with HSA-based hydrogels in the presence and absence of phage PA57. In the control group (hydrogel without phage), robust bacterial population growth was observed: the optical density of the culture increased progressively with incubation time, reaching OD_600_ values of approximately 3.5 by the end of the experiment (48 h) (Figure 12). This indicates progression into the stationary phase and confirms the absence of intrinsic antimicrobial activity of the protein matrix. In contrast, hydrogel samples loaded with bacteriophage PA57 demonstrated pronounced antibacterial activity during the early and middle stages of incubation. Bacterial growth was effectively suppressed for the first 30 h, maintaining low OD_600_ values (0.2–0.5) and representing a 5- to 10-fold reduction compared with the control. The late-stage regrowth may be explained by several non-mutually exclusive mechanisms, including the emergence of phage-resistant bacterial subpopulations, progressive depletion of infectious phage particles below the threshold required for sustained suppression, or adaptation of the bacterial culture to phage predation pressure. Nevertheless, the final bacterial load in the PA57 group remained lower than in the control. These findings confirm that the incorporation of bacteriophage PA57 into the HSA-based hydrogel matrix preserves its lytic activity and ensures effective initial suppression and significant delay of *P. aeruginosa* growth in suspension culture, which is a prerequisite for the application of this platform in the treatment of infected wounds.

### 2.3. Evaluation of Cytocompatibility and Cell Behavior on HSA-Based Hydrogels

For comprehensive assessment of the developed HSA-based hydrogel as a wound dressing, biocompatibility studies were conducted using two cell lines: HaCaT (human keratinocytes) and MRC-5 (human lung fibroblasts). The selection of these cell lines is determined by their role in the wound healing process: keratinocytes ensure wound reepithelialization, while fibroblasts are responsible for granulation tissue formation.

#### 2.3.1. Morphological Analysis and Cell Adhesion

**Figure 13:**
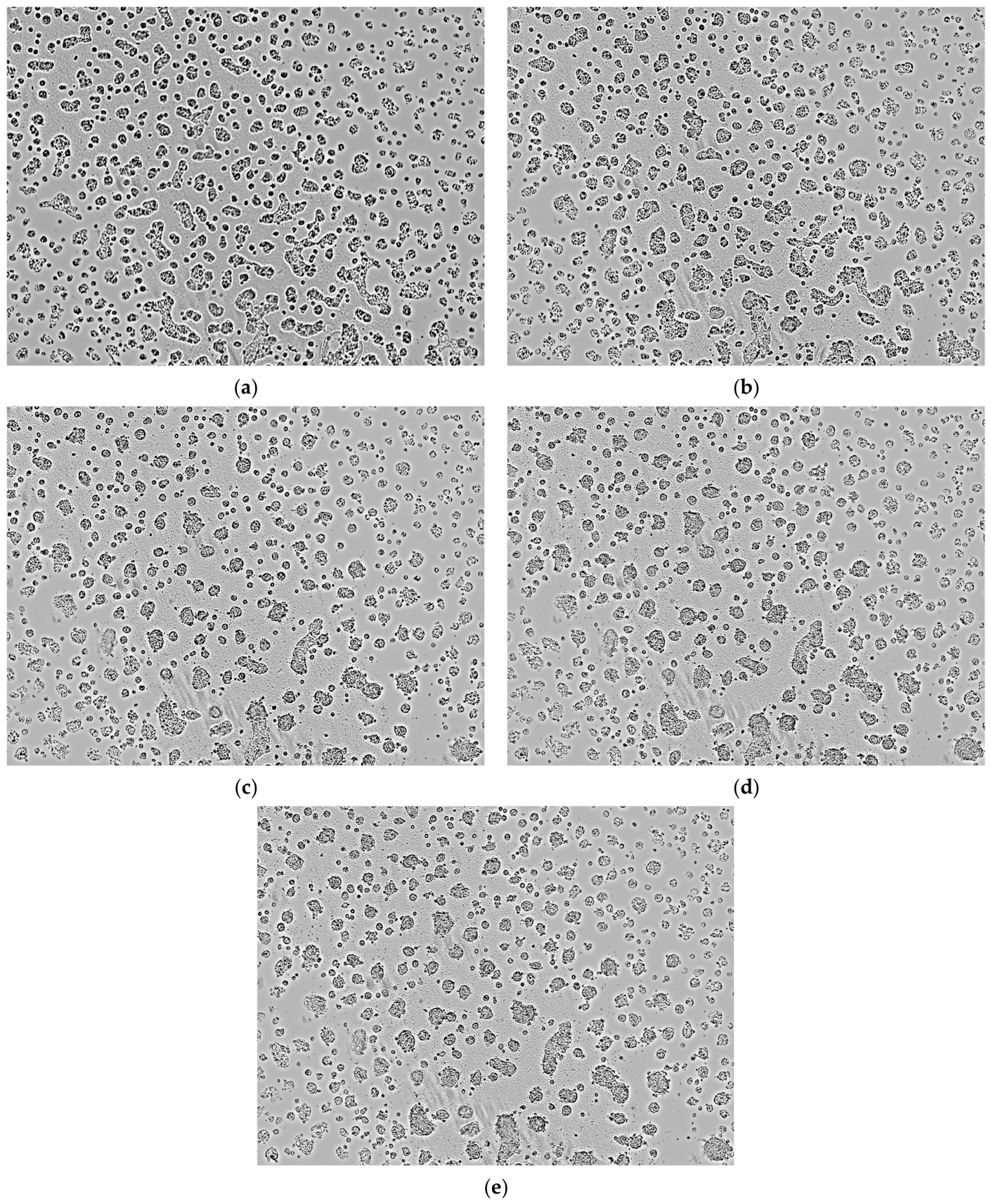
Morphology of HaCaT cells cultured on HSA-based hydrogels: (**a**) 0 h, (**b**) 6 h, (**c**) 12 h, (**d**) 18 h, and (**e**) 24 h.

Analysis of microscopic images of the HaCaT cell line over 0–24 h (Figure 13) showed that at the initial stage of cultivation, cells exhibit characteristic flattened polygonal morphology with well-defined adhesive contacts, indicating successful primary adhesion to the hydrogel surface. During the 6–18 h interval, changes in cell morphology were observed: cells acquired a rounded shape, which may be attributed to adaptation to the presence of ethanol in the hydrogel composition. By 24 h, cells again demonstrated spread morphology similar to the initial state, indicating preservation of viability and the ability to re-adhere after the adaptation period.

**Figure 14:**
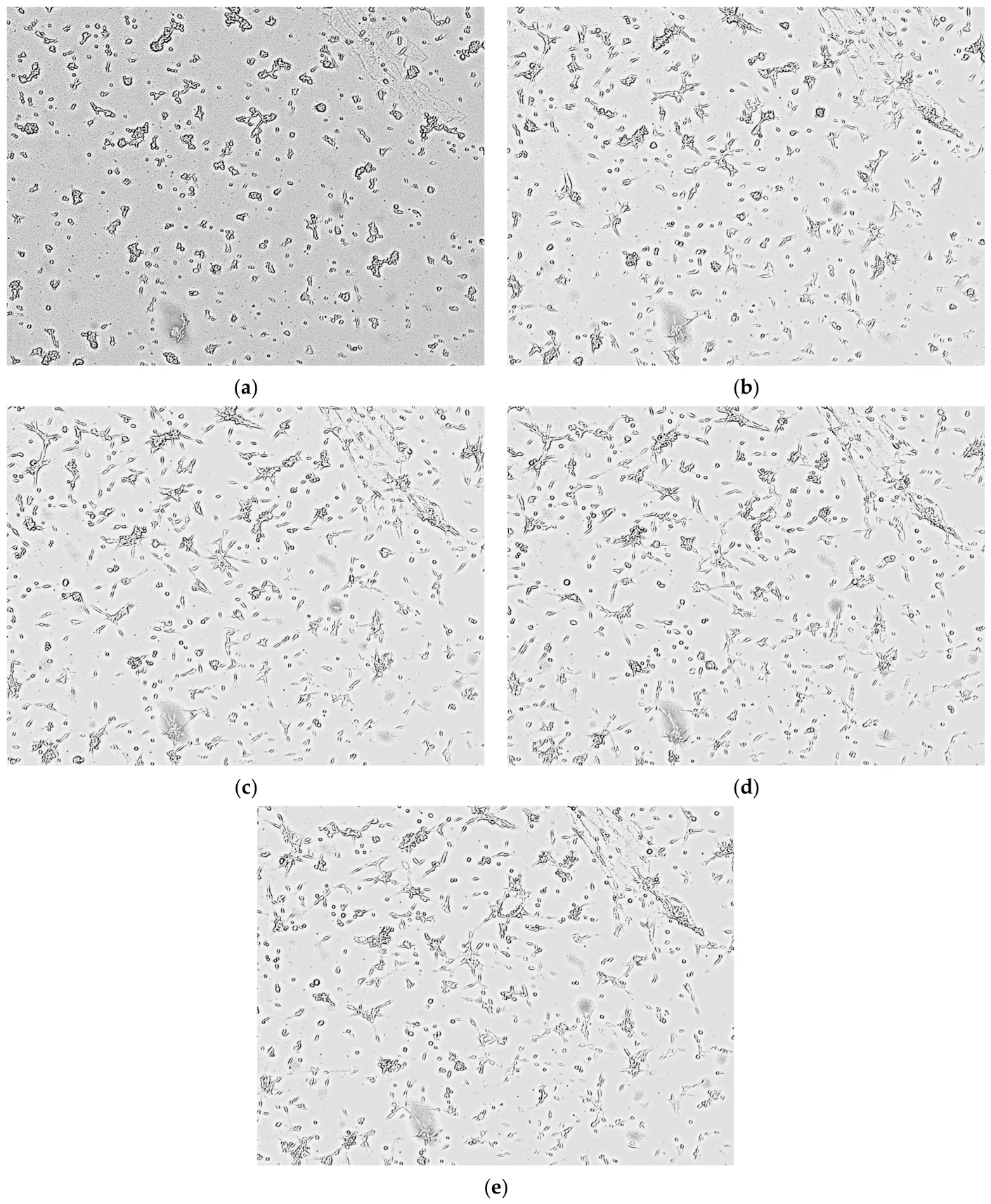
Morphology of MRC-5 cells cultured on HSA-based hydrogels: (**a**) 0 h, (**b**) 6 h, (**c**) 12 h, (**d**) 18 h, and (**e**) 24 h.

MRC-5 cells exhibited different behavioral dynamics (Figure 14). At the initial stage (0 h), cells were predominantly present as rounded aggregates. By 6 h, cells actively attached to the surface, acquiring morphology characteristic of fibroblasts—spindle-shaped and stellate (star-like) with extended cytoplasmic processes. During the 12–24 h interval, cells fully spread, forming a branched network, which indicates a healthy phenotype. Such morphology is characteristic of healthy fibroblasts with well-developed cytoskeletal structures, which are prerequisites for potential migration and extracellular matrix synthesis.

#### 2.3.2. MTT Assay

**Figure 15:**
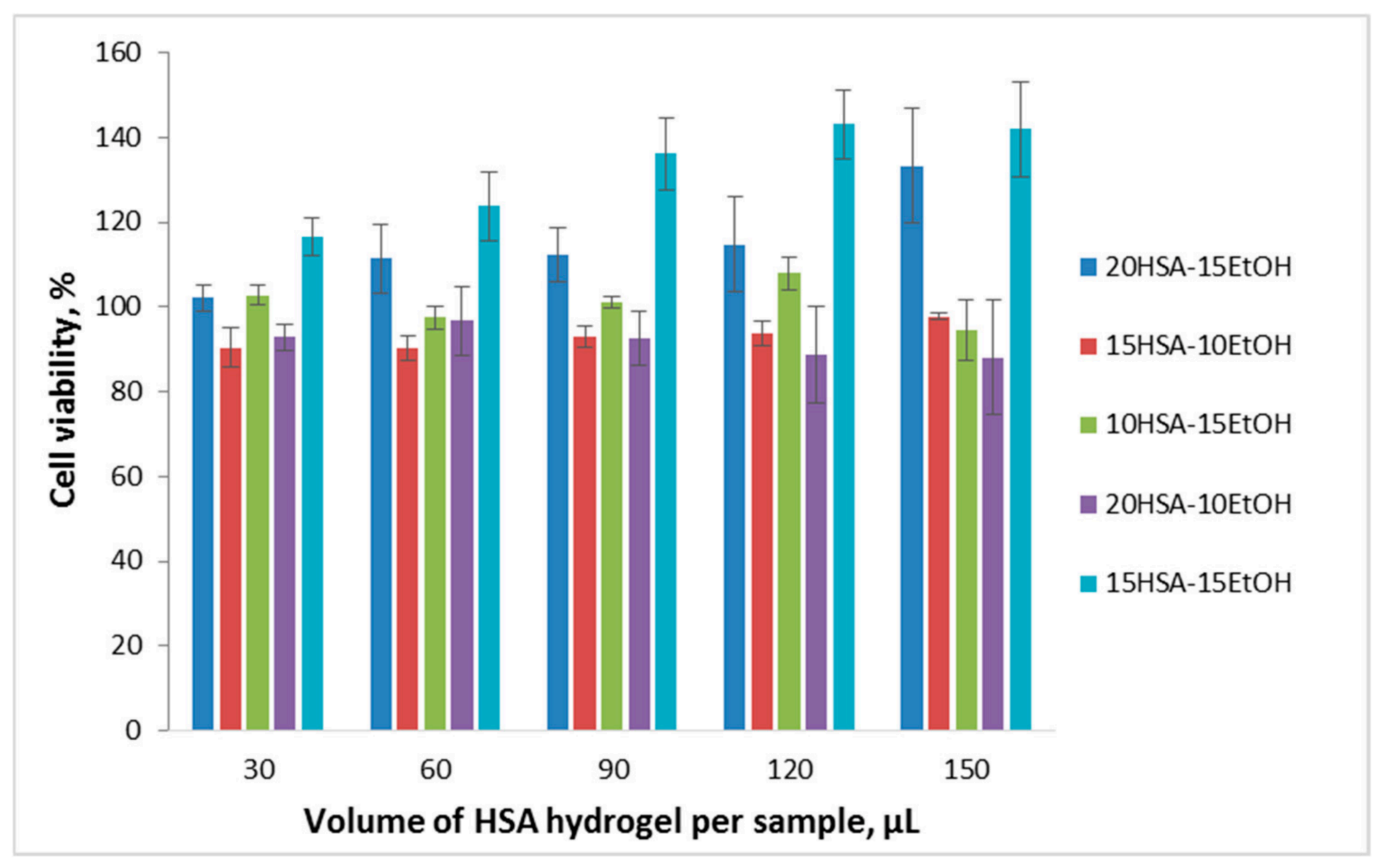
HaCaT cell viability assessed by the MTT assay after incubation with HSA-based hydrogels. Tested formulations: 20HSA-15EtOH, 15HSA-10EtOH, 10HSA-15EtOH, 20HSA-10EtOH, and 15HSA-15EtOH (HSA, % (*w*/*v*); EtOH, % (*v*/*v*)).

The Figure 15 presents cytotoxicity assessment data for HaCaT keratinocyte cell line incubated with hydrogels synthesized from HSA and ethanol in various ratios. The MTT assay results demonstrate that all tested samples exhibit low cytotoxicity. At all measurement time points, cell viability remains high (predominantly in the range of 80% to 140% compared with the control). Values exceeding 100% may indicate not only the absence of a toxic effect but also possible stimulation of cell proliferation. The enhanced survival of keratinocytes upon addition of 20HSA-15EtOH and 15HSA-15EtOH hydrogels can be explained by the increased stiffness of these gels compared with other formulations. This stiffness may serve as an extracellular matrix, providing cells with additional space for proliferation.

**Figure 16:**
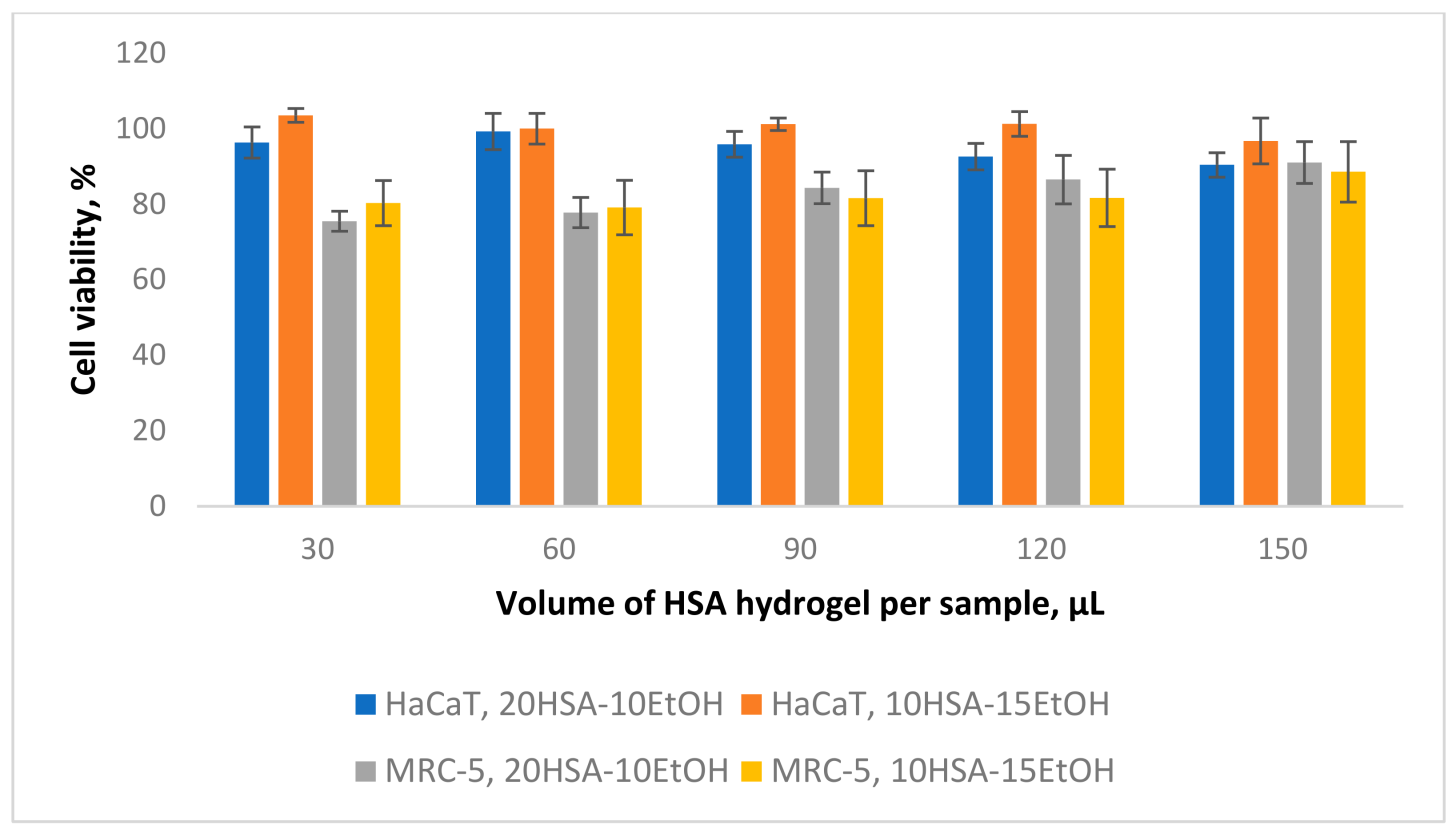
HaCaT and MRC-5 cell viability assessed by the MTT assay after incubation with HSA-based hydrogels. Cell viability was evaluated for two hydrogel formulations: 20HSA-10EtOH and 10HSA-15EtOH (HSA, % (*w*/*v*); EtOH, % (*v*/*v*).

When comparing the viability of HaCaT keratinocytes and MRC-5 fibroblasts (Figure 16), fibroblast viability was slightly lower (75–85% relative to the control) but remained stable across all hydrogel volumes without any decreasing trend. This difference between the cell lines may be attributed to cell type-specific metabolic features, as well as varying sensitivities to the hydrogel components; however, these assumptions are speculative and will not be investigated in detail in the present study. Furthermore, increasing the ethanol concentration from 10% to 15% (*v*/*v*) (with a simultaneous decrease in HSA concentration from 20% to 10% *w*/*v*) did not exert a statistically significant effect on the viability of any of the studied cell lines. This suggests that serum albumin mitigates the potential cytotoxic effects of ethanol, maintaining a favorable microenvironment for the cells. Thus, the human serum albumin-based hydrogel demonstrates excellent biocompatibility with the key cells involved in wound healing and can be recommended for further studies as a promising wound dressing.

## 3. Conclusions

In this study, we demonstrate for the first time the successful incorporation of the lytic bacteriophage PA57 into HSA-based hydrogels fabricated via a combined thermal- and ethanol-induced gelation method. Comprehensive characterization of the developed systems revealed that despite the sensitivity of PA57 to the synergistic effects of elevated temperature and ethanol, the HSA protein matrix is capable of enhancing the preservation of viral particles during hydrogel formation.

HSA concentration was identified as a critical parameter governing both the preservation of phage infectivity and the release profile. Increasing the protein content from 10% to 20% (*w*/*v*) increased the yield of viable phage particles from 1.3 ± 0.6% to 10.3 ± 2.2% of the nominal loading. Although the mechanism of this effect remains to be elucidated, these data are consistent with the hypothesis that a denser albumin network provides a more favorable microenvironment limiting virion inactivation.

Kinetic analysis revealed qualitative differences in release mechanisms depending on the polymer network density. Hydrogels with lower HSA concentrations (10–15% *w*/*v*) exhibited a pronounced initial burst release within the first 6 h, followed by a gradual decline in the release rate. In contrast, denser matrices (20% *w*/*v* HSA) provided a sustained release profile, characterized by a less pronounced initial burst and a delayed release peak observed at 24 h. Quantitative modeling of the 20HSA-15EtOH formulation demonstrated that the Korsmeyer–Peppas equation provides the best fit to the experimental data (R^2^ = 0.9957). The calculated release exponent (n = 0.61) indicates an anomalous transport mechanism, suggesting that phage release from the dense protein matrix is governed by a combination of Fickian diffusion and gradual matrix relaxation or swelling. This characteristic renders the 20% (*w*/*v*) HSA formulation particularly promising for the development of long-acting therapeutic systems.

Rheological assessments confirmed that all developed formulations exhibit pseudoplastic (shear-thinning) behavior, facilitating easy application to wound surfaces under mechanical stress while maintaining structural integrity at rest. The incorporation of bacteriophage did not significantly alter the rheological properties of the matrices, confirming their structural compatibility.

The functional activity of the incorporated phage was confirmed through a series of in vitro assays. The spot lysis assay on a bacterial lawn demonstrated the preservation of the lytic activity of the released particles, while suspension culture experiments revealed pronounced suppression of *P. aeruginosa* growth compared with the control. In addition to their antimicrobial properties, the hydrogels demonstrated excellent cytocompatibility and supported the proliferative activity of human keratinocytes (HaCaT) and fibroblasts (MRC-5), which is of fundamental importance for the development of wound healing formulations.

While the current study did not directly evaluate combination therapy, the developed HSA-based platform demonstrates the physicochemical compatibility necessary for future co-delivery of bacteriophages and low-molecular-weight antibiotics. The hypothesized synergistic action of phage and antibacterial therapy, coupled with controlled release from a biocompatible HSA matrix, could theoretically help overcome key limitations of phage therapy, including rapid virion clearance from the wound bed, insufficient penetration into bacterial biofilms, and the emergence of resistance. However, these potential advantages remain to be experimentally validated in future preclinical studies specifically designed to assess combination regimens.

## 4. Materials and Methods

### 4.1. Materials

Lyophilized human serum albumin (HSA; REANHAL, Budapest, Hungary) was used without further purification. Ethanol (EtOH; Kemerovo Pharmaceutical Plant, Kemerovo, Russian Federation) was dehydrated by laboratory-scale rectification to obtain anhydrous grade. Type 1 ultrapure water was obtained using a Milli-Q water purification system (Merck Millipore, Billerica, Massachusetts, USA). Dulbecco’s phosphate-buffered saline (PBS; pH 7.4) in tablet form was purchased from Sigma-Aldrich (St. Louis, MO, USA).

### 4.2. Bacteriophages

#### 4.2.1. Source of Bacteriophage and Host Bacterial Strain

Both bacteriophage PA57 and its host—the extensively drug-resistant (XDR) strain Pseudomonas aeruginosa CEMTC 670—were obtained from the Collection of Extremophilic Microorganisms and Type Cultures of the Institute of Chemical Biology and Fundamental Medicine, Siberian Branch of the Russian Academy of Sciences (CEMTC ICBFM SB RAS).

#### 4.2.2. Bacteriophage Propagation

Long-term storage of bacterial strains was achieved at −80 °C using 25% (*v*/*v*) glycerol. Experimental cultures were grown either in liquid Nutrient Broth (CondaLab, Madrid, Spain) or on solid Nutrient Broth Agar (Microgen, Moscow, Russia). Phage PA57 amplification was performed by infecting a 50 mL mid-logarithmic culture of *P. aeruginosa* CEMTC 670 (OD_600_ = 0.3–0.4) at an MOI of 0.1. Following infection, the culture was shaken at 37 °C until total bacterial lysis was achieved. The lysate was then cleared of cellular remnants via centrifugation (10,000× *g*, 30 min). To obtain a sterile phage suspension, the supernatant was filtered through a 0.22 µm pore size syringe filter (Nest, Wuxi, China). The final phage titer was assessed by spotting 5 µL of serial tenfold dilutions onto a fresh host lawn. The resulting high-titer lysate was utilized in all further experimental procedures.

#### 4.2.3. Plaque Assay

The morphological characteristics of bacteriophage PA57 were evaluated using the spot assay method [26]. *P. aeruginosa* CEMTC 670 served as the host culture. Serial dilutions of the PA57 phage lysate (titers: 10^2^, 10^3^, and 10^4^ plaque-forming units per milliliter, PFU/mL) were spotted onto the surface of agarized nutrient medium overlaid with a bacterial lawn. Plates were incubated overnight at 37 °C, followed by visual assessment of plaque morphology and measurement of plaque diameters. Plaque diameters were quantified using ImageJ software (version 1.53t, National Institutes of Health, Bethesda, MD, USA) based on standardized digital images acquired under uniform illumination conditions. Mean plaque size and standard deviation were calculated using appropriate statistical methods.

#### 4.2.4. Stability of Phage Particles

The stability of bacteriophage PA57 in media mimicking the conditions of HSA-based hydrogel formation was evaluated in vitro. As test systems, we used HSA solutions in phosphate-buffered saline (PBS) (final protein concentration up to 20% *w*/*v*) and mixtures of PBS with ethanol (ethanol volume fraction up to 50%). A phage suspension with an initial titer ≥ 10^9^ plaque-forming units per milliliter (PFU/mL) was added to the test solutions to achieve a final concentration of at least 10^8^ PFU/mL. To simulate the thermally induced gelation stage, samples were incubated at 60 °C for 15 min, followed by incubation at 25 °C for 24 h under constant agitation (300 rpm). A control consisted of a phage suspension in pure PBS subjected to an identical temperature regimen. Upon completion of the experiment, the infectious titer of virions was determined using the double agar overlay method according to Gracia on a lawn of the susceptible *P. aeruginosa* CEMTC 670 culture. Phage stability was assessed based on the degree of preservation of the initial infectious titer relative to the control sample. All experiments were performed in three biological replicates.

#### 4.2.5. Assessment of Bacteriophage Lytic Activity

The one-step growth curve of bacterial lysis throughout the life cycle of bacteriophage PA57 was determined as previously described [27]. Briefly, PA57 phage particles were added to an exponentially growing culture of *P. aeruginosa* CEMTC 670 at a multiplicity of infection (MOI) of 0.1. Infected cultures were incubated with shaking at 37 °C for 5 h, with aliquots collected every 30 min to determine bacterial titers. Based on the obtained data, a one-step growth curve was constructed. Experiments were conducted in two biological replicates, each comprising three technical replicates.

#### 4.2.6. Phage Genome Sequencing and Analysis

Phage DNA purification was performed as described previously with minor modifications [28]. In brief, phage suspension was mixed with a DNase buffer (Evrogen, Moscow, Russia). DNase I and RNase (both from Evrogen, Moscow, Russia) were then added to the mixture at a final concentration of 5 µg/mL each. The phage-containing solution was incubated at 37 °C for 30 min. Next, EDTA (pH 8.0), SDS, and proteinase K (Thermo Fisher Scientific, Waltham, Massachusetts, USA) were added to final concentrations of 20 mM, 0.5%, and 150 µg/mL, respectively. The phage-containing mixture was incubated at 55 °C for 3 h. After that, the PA57 DNA was purified using phenol/chloroform extraction. To precipitate the DNA, 96% ethanol was used, supplemented with 1/30 volume of 3 M sodium acetate (pH 5.1).

A paired-end sequencing library was prepared using NEBNext Ultra II DNA Library Prep Kit for Illumina and Multiplex Oligos for Illumina Dual Index Primers Set 1 (New England Biolabs, Inc., Ipswich, MA, USA) for further sequencing on the MiSeq sequencer with a MiSeq reagent kit v.2 (2 × 250-cycles) (Illumina, San Diego, CA, USA). The phage genome de novo assembly was performed using SPAdes v.3.0 (http://cab.spbu.ru/software/spades, accessed on 18 November 2024), resulting in one main circular contig with a coverage of 46.69. Pharokka (accessed on April 20, 2026) was applied for the PA57 genome annotation. The search for virulence factors and antibiotic resistance genes was carried out using the Virulence Factor (VR) database (http://www.mgc.ac.cn/VFs, accessed on 22 April 2026) and Antibiotic Resistance Gene (AGR) database (https://card.mcmaster.ca/rgi, accessed on 22 April 2026), respectively. BACPHLIP software [29] was used to predict lifestyle for the phage PA57. The genome of PA57 was deposited to the NCBI GenBank database with the accession number PZ520287.

To assess the taxonomy of phage PA57, a comparative proteomic phylogenetic anal-ysis was performed using Viral Proteome Tree Server (ViPTree) [30] (https://www.genome.jp/viptree, accessed 23 April 2026).

### 4.3. Hydrogels

#### 4.3.1. Preparation of Human Serum Albumin-Based Hydrogels Loaded with Bacteriophage PA57

HSA-based hydrogels were prepared using a combined thermal- and ethanol-induced gelation method, as previously described [23]. Hydrogel composites based on HSA with immobilized bacteriophage PA57 were fabricated via the same combined thermo- and ethanol-induced gelation protocol. Lyophilized HSA was dissolved in phosphate-buffered saline (PBS, pH 7.4) in 2 mL microcentrifuge tubes; the buffer volume was calculated to account for the subsequent addition of phage lysate to maintain the final concentration of all components. Following complete protein dissolution, a calculated volume of absolute ethanol was added to the solution to achieve a final ethanol volume fraction of 15–20%. A suspension of bacteriophage PA57 with an initial titer ≥ 1 × 10^9^ PFU/mL was introduced into the reaction mixture to reach a final concentration of at least 1 × 10^8^ PFU/mL. Gelation was induced by static heating of the homogenized mixture at 60 °C for 15 min.

#### 4.3.2. Electrophoretic Analysis of Human Serum Albumin

The protein composition of HSA-based hydrogels was analyzed by sodium dodecyl sulfate–polyacrylamide gel electrophoresis (SDS-PAGE) under denaturing conditions, following the standard Laemmli protocol. Hydrogel samples were solubilized in sample loading buffer (62.5 mM Tris-HCl, pH 6.8, 2% SDS, 10% glycerol, 5% β-mercaptoethanol, 0.01% bromophenol blue) and denatured at 95 °C for 5 min. Protein separation was performed in a vertical electrophoresis system using a stacking gel (4% acrylamide) and a resolving gel (12% acrylamide) under constant voltage (120 V). Following electrophoresis, gels were stained with Coomassie Brilliant Blue R-250 to visualize protein bands. Molecular weights of proteins were determined by comparison with the mobility of standard protein markers (premixed ladder, range 10–250 kDa).

#### 4.3.3. Measurement of Dynamic Viscosity of HSA-Based Hydrogels

Rheological measurements of dynamic viscosity were conducted at 25 °C using a Brookfield DV3T-RV rotational viscometer (Middleboro, MA, USA) with a cone-and-plate configuration, controlled via RheocalcT software (v1.2.19). Appropriate spindles (CPE-40 or CPE-52) were applied according to the viscosity of the tested samples.

#### 4.3.4. Evaluation of the Lytic Activity of HSA–Phage Hydrogel Formulations Against *P. aeruginosa*

The antibacterial activity of the HSA-based hydrogels was assessed using an agar diffusion assay. Sterile Petri dishes were prepared with a basal layer of nutrient agar (Microgen, Nizhny Novgorod, Russia). A top layer of LB agar (Servicebio, Wuhan, China), inoculated with a standardized suspension of *P. aeruginosa* (~1 × 10^6^ CFU/mL), was subsequently poured over the base. Approximately 70 μL of each hydrogel formulation was carefully dispensed onto the agar surface. All plates were incubated at 37 °C for 18–24 h in a temperature-controlled incubator.

#### 4.3.5. Evaluation of Phage Release Kinetics from Human Serum Albumin-Based Hydrogel Formulations

The release kinetics of bacteriophage PA57 from the hydrogel matrices were investigated in vitro under dynamic incubation conditions. Hydrogel samples (250 μL) containing phage at a titer ≥ 1 × 10^8^ PFU/mL were prepared in 2 mL microcentrifuge tubes (Eppendorf-type), and 750 μL of phosphate-buffered saline (PBS, pH 7.4) was added to each tube. Incubation was carried out at 37 °C on a temperature-controlled orbital shaker at 300 rpm to simulate physiological conditions and ensure homogeneous mass transfer. At predetermined time intervals, 550 μL aliquots were withdrawn from the supernatant layer above each hydrogel sample and immediately replaced with an equal volume of fresh PBS to maintain sink conditions. The concentration of released phage particles in each aliquot was quantified using the double agar overlay plaque assay according to Gratia, using *P. aeruginosa* CEMTC 670 as the indicator strain. Similarly, the release kinetics of phage PA57 from the 20HSA-15EtOH matrix was investigated; however, the initial phage loading in this case was ≥1 × 10^9^ PFU/mL.

Kinetic modeling of phage PA57 release from the 20HSA-15EtOH matrix was performed using four kinetic models (zero-order, first-order, Higuchi, and Korsmeyer–Peppas) in Microsoft Excel through the linearization of the release kinetic data. The cumulative release at the 46 h time point was taken as M∞.

#### 4.3.6. Determination of Bacteriophage Titer Released upon Complete Dispersion of the Hydrogel Matrix

To assess the maximum quantity of bacteriophage potentially available for release upon complete disruption of the hydrogel matrix, experiments involving forced dispersion of samples were conducted. In a 2 mL microcentrifuge tube, 250 µL of PA57-loaded hydrogel was prepared (100 µL of phage suspension with a titer of 2 × 10^8^ PFU/mL was added to each hydrogel). After hydrogel formation, 750 µL of phosphate-buffered saline (PBS, pH 7.4) was added on top, and the resulting mixture was vortexed until a relatively homogeneous suspension was obtained. The concentration of released phage particles in the resulting samples was determined using the double agar overlay plaque assay according to Gratia on a lawn of the susceptible *P. aeruginosa* CEMTC 670 strain. Phage titers were expressed as plaque-forming units per milliliter (PFU/mL) and normalized to the theoretical initial phage loading to determine phage survival during hydrogel formation.

#### 4.3.7. Investigation of Lytic Activity of Phage-Immobilized Hydrogels In Vitro in Suspension Culture

The antibacterial efficacy of hydrogels containing immobilized bacteriophage PA57 was evaluated in vitro under suspension culture conditions. Hydrogel samples (600 µL; initial phage titer ≥ 1 × 10^8^ PFU/mL), prepared in sterile 15 mL Falcon-type conical tubes, were incubated with 2000 µL of exponentially growing *P. aeruginosa* CEMTC 670 culture (titer ≈ 1 × 10^6^ CFU/mL). The reaction mixture was incubated at 37 °C with constant agitation (300 rpm) on a temperature-controlled orbital shaker. The kinetics of bacterial lysis were monitored by measuring the optical density of the suspension at λ = 600 nm. As a negative control, phage-free hydrogel incubated with bacterial culture was used.

#### 4.3.8. Cytotoxicity Study of HSA-Based Hydrogels

The cytotoxicity of HSA-based hydrogels was evaluated using the colorimetric MTT (3-(4,5-dimethylthiazol-2-yl)-2,5-diphenyltetrazolium bromide) assay. Experiments were conducted on two cell lines: the MRC-5 human diploid lung fibroblast cell line and the HaCaT human keratinocyte cell line derived from normal epidermis. The cytotoxicity evaluation for the MRC-5 cell line was performed according to a previously described protocol [23]. The MTT assay protocol for HaCaT cells was identical, with the exception that RPMI 1640 medium (Thermo Fisher Scientific, Waltham, MA, USA) was used as the culture medium.

#### 4.3.9. Biocompatibility Assessment of Human Serum Albumin-Based Hydrogels

To evaluate cell adhesion, morphological dynamics, and proliferative activity on the hydrogel surface, two cell lines were used: the MRC-5 and the HaCaT. The procedure for dish preparation and cell seeding was performed according to a previously described protocol [23]. The dynamics of cell attachment, morphological changes, and behavioral responses were monitored in real time using the JuLI Stage JS1000S automated live-cell imaging system (NanoEntek, Seoul, South Korea), with images acquired at predetermined time intervals (0, 6, 12, 18, and 24 h) without removing the samples from the incubator.

#### 4.3.10. Statistical Analysis

All experiments were performed with at least three biological replicates, each comprising at least two technical replicates. Data are presented as mean ± SD. Data on the suppression of bacterial growth in suspension culture were analyzed using GraphPad Prism 8.0.2 software via two-way analysis of variance (two-way ANOVA; factors: treatment (presence/absence of phage PA57) and time), followed by Sidak’s post hoc test (α = 0.05; one family of 5 comparisons) to compare the PA57 and control groups at each time point, controlling for the family-wise error rate.

## Figures and Tables

**Table 1:** Effect of PA57 loading concentration on the lytic efficacy of HSA-based hydrogels against *P. aeruginosa*.

Initial Phage Loading (PFU/mL)	Lysis Zone View
1 × 10^8^	Complete, smooth margins
1 × 10^7^	Complete, irregular margins
1 × 10^6^	Complete, irregular margins
1 × 10^5^	Partial lysis
1 × 10^4^	Partial lysis
1 × 10^3^	No lysis

**Table 2:** Calculated total recovery of phage PA57 following complete dispersion of HSA-based hydrogel formulations with varying protein content (10–20% (*w*/*v*) HSA) and constant ethanol concentration (15% (*v*/*v*)). Initial phage loading: 2 × 10^8^ PFU/mL.

Formulation	Retained Phage Content in the Gel, %
15HSA-15EtOH	3.3 ± 1.5
20HSA-15EtOH	10.3 ± 2.2

**Table 3:** Coefficients of determination (R^2^) for kinetic release models of PA57 from 20HSA-15EtOH hydrogel.

Kinetic Model	Equation of Model After Linearization	Coefficient of Determination, R^2^
Zero-order	$\frac{M_{t}}{M_{\infty }}=k_{0}\cdot t$	0.7584
First-order	$\ln\left(M_{\infty }-M_{t}\right)=ln(M_{\infty })-k_{1}\cdot t$	0.8801
Higuchi	$\frac{M_{t}}{M_{\infty }}=a+k_{H}\cdot \sqrt{t}$	0.8903
Korsmeyer–Peppas	$ln(\frac{M_{t}}{M_{\infty }})={ln(k}_{KP})+n\cdot ln(t)$	0.9957

## Data Availability

The data presented in this study are openly available in article.

## Abbreviations

The following abbreviations are used in this manuscript:

HSA	human serum albumin
EtOH	ethanol
15HSA-15EtOH	hydrogel 15% (*w*/*v*) HSA-15% (*v*/*v*) EtOH
10HSA-15EtOH	hydrogel 10% (*w*/*v*) HSA-15% (*v*/*v*) EtOH
20HSA-15EtOH	hydrogel 20% (*w*/*v*) HSA-15% (*v*/*v*) EtOH
20HSA-10EtOH	hydrogel 20% (*w*/*v*) HSA-10% (*v*/*v*) EtOH
15HSA-10EtOH	hydrogel 15% (*w*/*v*) HSA-10% (*v*/*v*) EtOH
CFU	colony-forming unit
CEMTC	Collection of Extremophilic Microorganisms and Type Cultures
